# Office of Student Affairs: Engagement and Leadership Opportunities for Medical Students, Residents, and Fellows

**DOI:** 10.15766/mep_2374-8265.11093

**Published:** 2021-02-05

**Authors:** Sunny Nakae, Yolanda Haywood, Latanya J. Love, Pooja Kothari, Fidencio Saldaña, John Paul Sánchez

**Affiliations:** 1 Senior Associate Dean for Diversity, Equity, Inclusion and Partnership, and Associate Professor of Medical Education, California University of Science Medicine; 2 Senior Associate Dean for Diversity and Faculty Affairs (Interim), and Associate Dean for Student Affairs, George Washington School of Medicine; 3 Associate Dean for Admissions and Student Affairs, Associate Dean for Diversity and Inclusion, and Associate Professor of Pediatrics, McGovern Medical School, The University of Texas Health Sciences Center; 4 First-Year Internal Medicine Resident, Montefiore Medical Center; 5 Dean for Students and Assistant Professor of Medicine, Harvard Medical School; 6 Professor, University of New Mexico School of Medicine; Senior Advisor, Office for Diversity, Equity, and Inclusion, Health Sciences Center, University of New Mexico; Executive Director, Latino Medical Student Organization National Inc.; President, Building the Next Generation of Academic Physicians, Inc.

**Keywords:** Office of Student Affairs, Leadership, Academic Medicine, Career Development

## Abstract

**Introduction:**

Exposing trainees to roles within medical school offices is an important, but often overlooked, component of academic medicine career development. This module described the roles and responsibilities of staff within the Office of Student Affairs (OSA) and opportunities for trainees to become engaged, lead, and develop student affairs-related competencies.

**Methods:**

The 90-minute workshop was presented at three regional conferences at US medical schools between September and December 2019. Participants were medical students, residents, and fellows from multiple institutions. The workshop consisted of a didactic portion describing OSA responsibilities and guiding principles, reflection exercises to gauge learners' engagement with the OSA, and case discussions on how trainees have led scholarly student affairs-related projects.

**Results:**

Among 28 participants, over 90%, agreed that each of the workshop objectives was met. Using the Wilcoxon signed-rank test, there was a statistically significant increase (*p* < .001) in participants' confidence to “list skills to be an effective advisor in the OSA,” and, “Advocate for student issues through the OSA.”

**Discussion:**

Trainees not only have the opportunity to access services through the OSA, but also serve and develop foundational competencies to eventually serve in an OSA leadership position. This workshop provided trainees early exposure to OSA administration to realize a career in academic medicine beyond the faculty role.

## Educational Objectives

By the end of this activity, learners will be able to:

1. Describe the role and responsibilities of the Office of Student Affairs (OSA).
2. Define guiding principles, skills, and behaviors required of student affairs professionals.
3. Describe leadership opportunities for medical students, residents, and/or fellows through the OSA.
4. Link trainee engagement opportunities through the OSA to core competencies for leadership in academic medicine roles.

## Introduction

There is an expanding body of literature describing the need for the development of leadership skills among physician trainees during both undergraduate and graduate medical education.^1–3^ Most of this literature is focused on leadership and management skills needed to ensure high quality patient care. The development of leadership skills needed to ensure high quality medical education are equally as important in ensuring that our health education systems are prepared for the changing landscape of practicing medicine.

As the United States population becomes increasingly diverse, the need for diversity in leadership becomes more crucial. There are few current programs designed to foster the acquisition of skills needed to prepare for a career in student affairs for undergraduate medical education. The AAMC includes a one-page document on their *Careers in Medicine* website that introduces trainees to a career path in student affairs.^4^ There is no current literature describing how or why health care professionals choose roles in student affairs leadership. It could be inferred, however, that the lack of exposure and awareness to this career pathway results in fewer MD-bearing candidates, less diverse candidates, and candidates who are less prepared to fulfill student affairs leadership roles.

Building the Next Generation of Academic Physicians (BNGAP) was established in 2010 to develop diverse talent for academic medicine. BNGAP's workshops are specifically designed to raise awareness among medical students and residents for academic careers. Members of BNGAP's curriculum committee created this workshop, which was subsequently reviewed by a diverse team of students and faculty. The workshop has been continually revised to meet the needs of a diverse group of trainees, in particular medical students, but was also applicable for residents and fellows. The core elements of the workshop were a PowerPoint presentation, a group activity, and small- and large-group case reflection exercises. This workshop was centered on leadership opportunities within the Office of Student Affairs (OSA) and the professional skills that are foundational to related faculty competencies. The authorship team of the workshop have experience in student affairs roles at their respective institutions. The years of expertise of the group are reflected in the design of the workshop and its content.

This workshop utilized a framework from social cognitive career theory (SCCT).^5^ SCCT posits that learners must first perceive high self-efficacy in order to engage in a career pursuit.^6^ There are four components that are foundational to Bandura's theory of self-efficacy upon which SCCT is built: exposure, role modeling, mentoring, and observation.^7^ Faculty experts delivered the content and simultaneously served as role models. As students engage in opportunities with the OSA they will be able to observe and reflect on their experiences. This will, in turn, facilitate a growth in confidence to pursue leadership roles in academic medicine. This workshop had an interactive activity designed to link current activities in student affairs with faculty competencies, thereby facilitating increased self-efficacy.

## Methods

### Development

The workshop curriculum was developed using the six-step approach to curriculum development described by Kern to expose and prepare students, residents, and fellows for leadership roles in student affairs.^8^ In step 1, our problem identification and general needs assessment consisted of surveying trainees as to their academic medicine career interests and performing a literature review on leadership development for trainees. For step 2, we relied on the faculty leadership competencies determined by Lucas et al.^9^ and the AAMC Group on Student Affairs Performance Framework^10^ to determine core areas for trainees to develop through engagement in the OSA. In step 3, we determined our goals and objectives based on the literature review and coauthor collaboration. In step 4, our chosen educational strategies included a PowerPoint presentation, reflection exercises, interactive activity, and case discussion. In step 5, the workshop was implemented among a subset of attendees at a series of national conferences designed for medical students, residents, and fellows interested in leadership positions in academic medicine. In step 6, evaluation and feedback, pre- and postworkshop questionnaires were developed for each participant so the workshop creators could evaluate the design and content of the workshop. The institutional review board application covered three sites (Weill Cornell College of Medicine, University of Oklahoma College of Medicine, and McGovern Medical School).

### Implementation

The beginning of the workshop involved a preworkshop evaluation (Appendix A) that asked trainees questions to assess their demographic characteristics, prior knowledge of and experience with the OSA, and to evaluate the efficacy of the workshop. Following the preevaluation, a PowerPoint presentation was used as the guide for the workshop, including a short didactic portion detailing the professional tenets of student affairs (Appendix B). Woven into the short didactic portion was an interactive activity designed to actively engage participants in understanding the scope of student affairs practice using everyday scenarios that leaders may face (Appendix C). Participants were given brief issues or scenarios that are common in student affairs practice and instructed to identify the core domains or collaborative areas under which they fell. Participants were also asked to identify overlapping areas and scenarios for potential trainee engagement. Following the activity, the facilitator discussed areas of opportunity within the OSA for medical students and residents to engage, as well as identified the several achievable leadership competencies students can strengthen while participating in the OSA (Appendix D). Two cases were then presented and discussed as a means of inspiring further conversation in understanding the complexities and leadership competencies in the OSA (Appendix E). The facilitators then shared customized career pathway slides and discussed their journeys into medical education leadership roles. Finally, a short question and answer session was conducted and the postworkshop survey (Appendix A) was handed out to provide an opportunity for trainees to discuss what they liked about the workshop and suggestions on how to improve the experience.

The workshop can be done by one or two facilitators who ideally are leaders in the OSA with experience in small-group facilitation. Instructors should allocate 3 hours to review the content of the training beforehand, including the facilitator guide (Appendix F), which served as the structure for the entirety of the workshop. In order to properly conduct the workshop facilitators will need: (1) markers, (2) wall space for the OSA duties activity (Appendix C), (3) audio/visual equipment for the PowerPoint presentation, (4) chairs and tables arranged to facilitate small-group discussion for three to seven participants, (5) printed copies of the core student affairs skills (Appendix D), cases (Appendix E), OSA duty activity scenarios (Appendix C), and evaluation forms (Appendix A). The suggested length of the workshop is 90 minutes, with a detailed outline below:

• Preworkshop evaluation: 2 minutes.
• Slides 1–4: 5 minutes.
• Slides 5–10: reflection and introduction, 15 minutes.
• Slide 11: OSA duties activity, 20 minutes.
• Slides 12–26: OSA core areas and values, 20 minutes.
• Slide 27–30: cases, 20 minutes.
• Slide 31–36: summary, questions, and wrap-up: 5 minutes.
• Postworkshop evaluation: 3 minutes.

This workshop was part of a series of BNGAP modules and can also be taught as singular sessions. Modifications to this workshop should remain anchored to the exposure, role modeling, mentoring, and observation framework upon which the curriculum was based. Leadership development through engagement with the OSA can pave the way for the next generation of rising leaders for academic medicine.

## Results

This workshop was implemented at three conference sites with a total of 35 participants: Weill Cornell College of Medicine, University of Oklahoma College of Medicine, and McGovern Medical School as a part of a regional BNGAP leadership conference. A total of 28 trainees completed partial or full workshop evaluations for the OSA workshop. The workshop was facilitated by a total of four presenters (one pair and two single presenters), including an assistant dean for diversity and inclusion, an associate professor of internal medicine, and two associate deans of student affairs.

Of the 28 attendees who responded to the preconference survey, 21 identified as medical students, two as residents, and two as fellows. The attendees hailed from five different states and the Dominican Republic.

Among the 28 respondents, two (7%) identified as Native American or Alaska Native, three (11%) as Asian, eight (29%) as Black or African-American, six (24%) as Hispanic or Latino, and nine (32%) as white. Thirteen (46%) identified as male, and 11 (39%) as female. Three (11%) identified as gay, lesbian, or bisexual. Demographic data were collected from participants as part of comprehensive evaluations for BNGAP careers in academic medicine conferences (where the workshops were offered). BNGAP's mission is to increase the diversity of the faculty and leadership of academic medicine.

Of attendees, 24 responded to the question, “How knowledgeable are you in identifying leadership opportunities for trainees to become engaged through the OSA?” In response, six attendees (21%) replied *not knowledgeable*, eight (29%) replied *somewhat knowledgeable*, nine (32%) replied *knowledgeable*, and one (4%) replied *very knowledgeable*. In assessing attendee background experience, they were also asked to identify if they have “participated on a committee or taskforce overseen by the OSA or its equivalent,” during several time periods. Ten (36%) reported experience prior to medical school and 14 (50%) during medical school. None of respondents reported experience during residency or after residency.

On the pre- and postworkshop survey, participants were asked to indicate on a Likert scale their level of confidence (0 = *no confidence*, 4 = *complete confidence*) regarding two student affairs-related tasks. For the statement, “List skills to be an effective advisor in the OSA”, the preworkshop mean and median values were 2.0 and 2.0, and postworkshop mean and median values were 3.4 and 4.0. For the statement, “Advocate for student issues through the OSA,” the preworkshop mean and median values were 2.4 and 3.0 and postworkshop mean and median values were 3.7 and 4.0. In applying the Wilcoxon signed-rank test there was a statistically significant difference in pre- and postworkshop survey responses for each question at *p* < .001.

Additionally, efficacy of the workshop was determined by asking attendees, “To what extent do you agree that the workshop learning objectives were met?” For all four objectives, over 90% of attendees either agreed or strongly agreed that the objectives were met.

Comments for the workshop were collected to identify positive areas of the curriculum, as well as suggestions on how to further improve the module. Overall, the participants found the workshop engaging, informative, and felt the cases were thought provoking. Several participants remarked that the workshop sufficiently detailed the different positions and associated responsibilities of the OSA: “I thoroughly enjoyed the explanation of the vast role of the OSA,” and, “I was familiar with the OSA, but the workshop enlightened me to all functions.” Respondents gained an appreciation of opportunities to become engaged through the OSA as illustrated in the following quotes: “Speaker gave wonderful examples of ways to get involved in student affairs on many levels, whether you're a first through fourth year,” and, “I learned a lot about the career trajectory for a role in student affairs.” Learners also valued facilitators' supportive comments regarding the role of students in the office with comments as follows: “The speaker's acknowledgement of the importance of the medical students input in this office,” and, “The speaker's advice about advocating for yourself and voicing desires through the office.”

In terms of how to improve the workshop, participants made several recommendations. One respondent recommended having speakers from within and outside of the home institution cofacilitate the session. Several respondents requested more specific examples of how trainees could become involved with student affairs-related committees, projects, and initiatives. Similarly, two respondents specifically requested a greater explanation of leadership opportunities through the office as featured through these quotes: “I believe that more time should have been spent discussing student leadership roles within the office and student-led publications/improvement projects,” and, “Better description of leadership opportunities for medical students and residents.”

## Discussion

Although trainees may be familiar with the OSA, as well as the services such offices provide to students, they may not see student affairs as a potential academic or career pursuit. The module presented information on the OSA and introduced opportunities for trainee involvement as well as potential career opportunities. In addition, it exposed students to diverse role models in academic medicine. Notably, only a small number of students expressed a high level of expertise in the area. Across all four learning objectives, over 90% of attendees across all three sites agreed or strongly agreed that the workshop objectives were met.

Through the presentation, cases, and duties activity, students were able to gain increasing awareness of the roles and responsibilities of the OSA. For educational objective 2, “Define guiding principles, skills, and behaviors required of student affairs professionals,” we provided the handout on specific faculty competencies developed by students through engagement with the OSA (Appendix D). Students were able to learn about leadership competencies as well as to reinforce material presented in the PowerPoint slides. This was meant to increase confidence in skills for future engagement, which was consistent with the SCCT framework that served as the scaffold for this workshop.

In the workshop feedback several respondents requested more specific examples of how trainees could become involved with student affairs-related committees, projects, and initiatives. Similarly, two respondents specifically requested a greater explanation of leadership opportunities through the office. Facilitators can personalize the presentation to include opportunities at their respective institutions and encourage students to seek out guidance from their home institutions. Furthermore, cofacilitating with faculty from the host institution may prove valuable, as specific examples can be provided.

Future directions for education modules in this area could include more specific learning activities that illustrate duties and skills of student affairs and faculty leadership. Policy change, advocacy, incident management, anti-racism, and career planning might all be specific workshops dedicated to linking trainee experiences with student affairs leadership and practice.

### Limitations

Although the participants came from different types of institutions and geographic regions, the sample size was limited and mainly consisted of medical students. The small number of residents and fellows among participants limited the strength of applicability to graduate medical education trainees. We did not receive comments indicating that the workshop was not received favorably by graduate medical education learners, but if this workshop were delivered to an exclusively graduate medical education audience, facilitators would need to take care to adapt and center the content on graduate medical education learners. The educational objectives were relevant to medical trainees of all levels, however more data are needed to ensure the information can be targeted to trainees in graduate medical education effectively.

The scope of the workshop and its length (90 minutes) created limitations in evaluating more complex outcomes across domains like self-efficacy, which is performative in nature. Following up with participants to assess their self-efficacy for engagement in student affairs at a later date might better help assess the effectiveness of the workshop and its aims. The data were also from one point in time; longitudinal follow-up would be useful in determining the impact of participation in student affairs-related projects in the future as well as eventual career choices.

As trainees consider academic medicine careers, this workshop effectively introduced them to possibilities within the OSA, a central office in all medical schools. More importantly, this workshop helped raise trainee awareness that beyond accessing services through the OSA, they can engage with, lead through, and build foundational leadership competencies through the OSA.

## Appendices

OSA Evaluation Forms.docxOSA PowerPoint.pptxOSA Duties Activity.docxOSA Chart.docxOSA Cases.docxOSA Facilitator Guide.docx
All appendices are peer reviewed as integral parts of the Original Publication.
