# Supplementary material for: Office of Student Affairs: Engagement and Leadership Opportunities for Medical Students, Residents, and Fellows
Source: MedEdPORTAL. 2021 Feb 5;17:11093. doi: 10.15766/mep_2374-8265.11093 (PMC7880253; doi:10.15766/mep_2374-8265.11093)
Supplement: Supplementary file 1 — OSA Evaluation Forms.docxOSA PowerPoint.pptxOSA Duties Activity.docxOSA Chart.docxOSA Cases.docxOSA Facilitator Guide.docx [file mep_2374-8265.11093-s001.zip › A. OSA Evaluation Forms.docx]

**Pre-Evaluation Survey**

| 1.How knowledgeable are you in identifying leadership opportunities for trainees to become engaged through the Office of Student Affairs (circle one) | Not Knowledgeable | | Somewhat Knowledgeable | | Knowledgeable | | Very knowledgeable | |
| --- | --- | --- | --- | --- | --- | --- | --- | --- |
| 2. Have you participated on a committee or taskforce overseen by the Office of Student Affairs or its equivalent during the following periods… (Y-Yes, N-No, NA – not applicable) | Prior to medical school  Y N NA | | During medical school or prior to residency  Y N NA | | During residency  Y N NA | | After residency  Y N NA | |
| **3. How much CONFIDENCE do you have in your ability to…** | **No Confidence**  **0** | **1** | | **2** | | **3** | | **Complete**  **Confidence**  **4** |
| 1. List skills to be an effective advisor in the Office of Student Affairs | 0 | 1 | | 2 | | 3 | | 4 |
| 1. Advocate for student issues through the Office of Student Affairs | 0 | 1 | | 2 | | 3 | | 4 |
| **DEMOGRAPHICS:** | | | | | | | | |
| 4. In which **STATE** is your medical school or residency program located? _____________________ | | | | | | | | |
| 5. Are you a (circle one):   1. Medical Student 2. Intern/Resident 3. Fellow 4. Other (please specify):_______________________________ | | | | | | | | |
| 6. What is your race/ethnicity (**circle all that apply)?**   1. American Indian or Alaska Native 2. Native Hawaiian or Other Pacific Islander 3. Asian 4. Black or African-American 5. Hispanic or Latino 6. White 7. Other (please specify): | | | | | | | | |
| 7. How do you self-identify (**circle one**)?   1. Straight or Heterosexual 2. Gay or Lesbian 3. Bisexual 4. Other (please specify): ____________________ | | | | | | | | |
| 8. How do you **self-identify**? Note: Respondents who self-identify as “Transgender female-to-male,” “Transgender male-to-female,” or “Transgender do not identify as exclusively male or female” are combined and displayed as “Transgender” (circle all that apply):   1. Male 2. Female 3. Transgender 4. Other: ________________________________ | | | | | | | | |

**Post-Evaluation Survey**

| **1. How much CONFIDENCE do you have in your ability to…** | **No Confidence**  **0** | **1** | **2** | **3** | **Complete**  **Confidence**  **4** |
| --- | --- | --- | --- | --- | --- |
| 1. List skills to be an effective advisor in the Office of Student Affairs | 0 | 1 | 2 | 3 | 4 |
| 1. Advocate for student issues through the Office of Student Affairs | 0 | 1 | 2 | 3 | 4 |
| **2. To what extent do you agree that the workshop learning objectives were met** | **Strongly agree** | **Agree** | **Neither agree or disagree** | **Disagree** | **Strongly disagree** |
| 1. Describe the roles and responsibilities of the Office of Student Affairs (OSA) | SA | A | N | D | SD |
| 1. Define guiding principles, skills, and behaviors required of student affairs professionals | SA | A | N | D | SD |
| 1. Describe leadership opportunities for medical students, residents, and/or fellows through the OSA | SA | A | N | D | SD |
| 1. Link student engagement opportunities through the OSA to core competencies for leadership in academic medicine roles | SA | A | N | D | SD |
| What did you like about this workshop? | | | | | |
| What suggestions do you have to improve this workshop? | | | | | |
