# Supplementary material for: Office of Student Affairs: Engagement and Leadership Opportunities for Medical Students, Residents, and Fellows
Source: MedEdPORTAL. 2021 Feb 5;17:11093. doi: 10.15766/mep_2374-8265.11093 (PMC7880253; doi:10.15766/mep_2374-8265.11093)
Supplement: Supplementary file 1 — OSA Evaluation Forms.docxOSA PowerPoint.pptxOSA Duties Activity.docxOSA Chart.docxOSA Cases.docxOSA Facilitator Guide.docx [file mep_2374-8265.11093-s001.zip › B. OSA PowerPoint.pptx]

## Slide 1
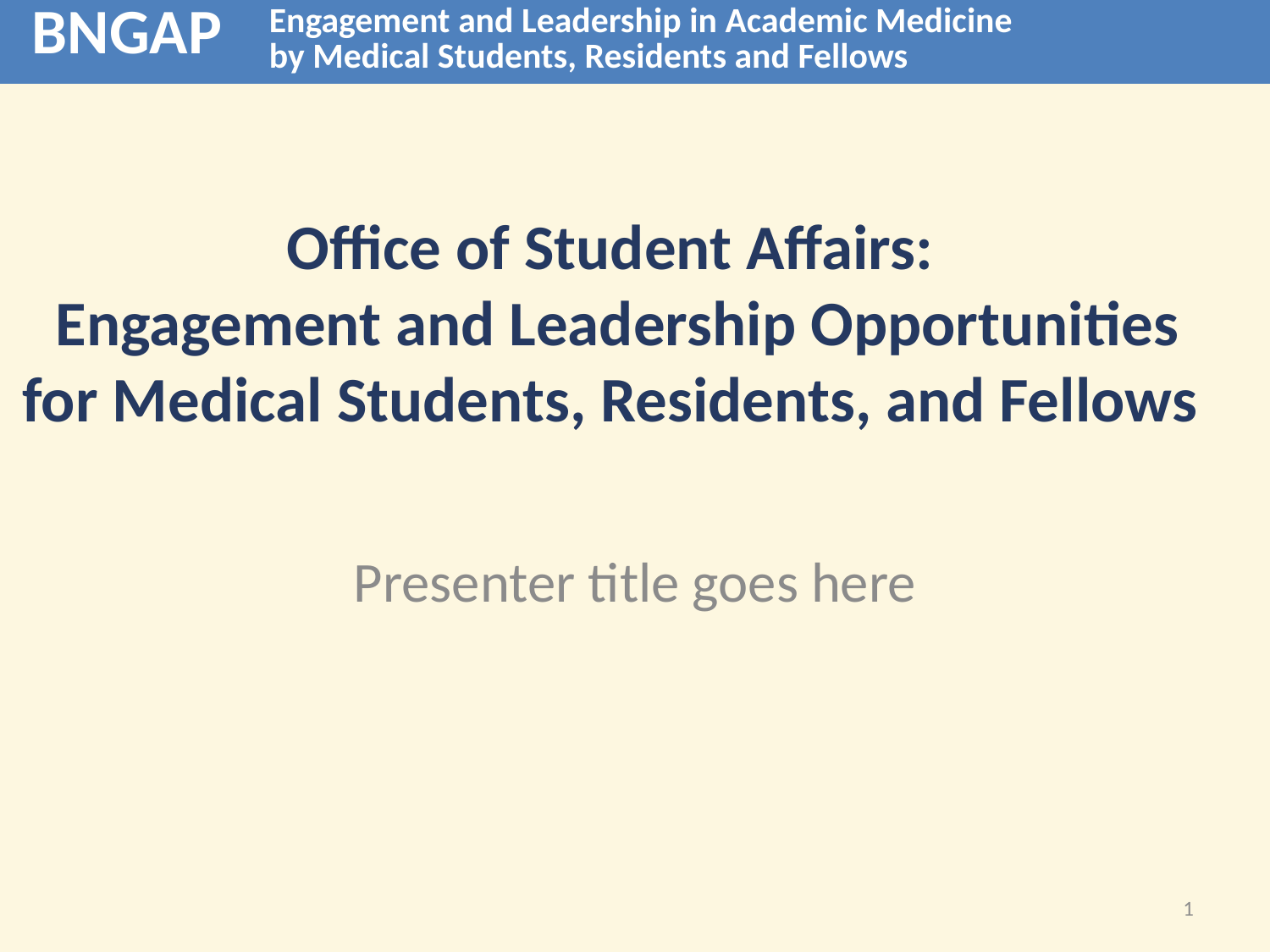

Office of Student Affairs:
Engagement and Leadership Opportunities for Medical Students, Residents, and Fellows
Presenter title goes here
1

## Slide 2
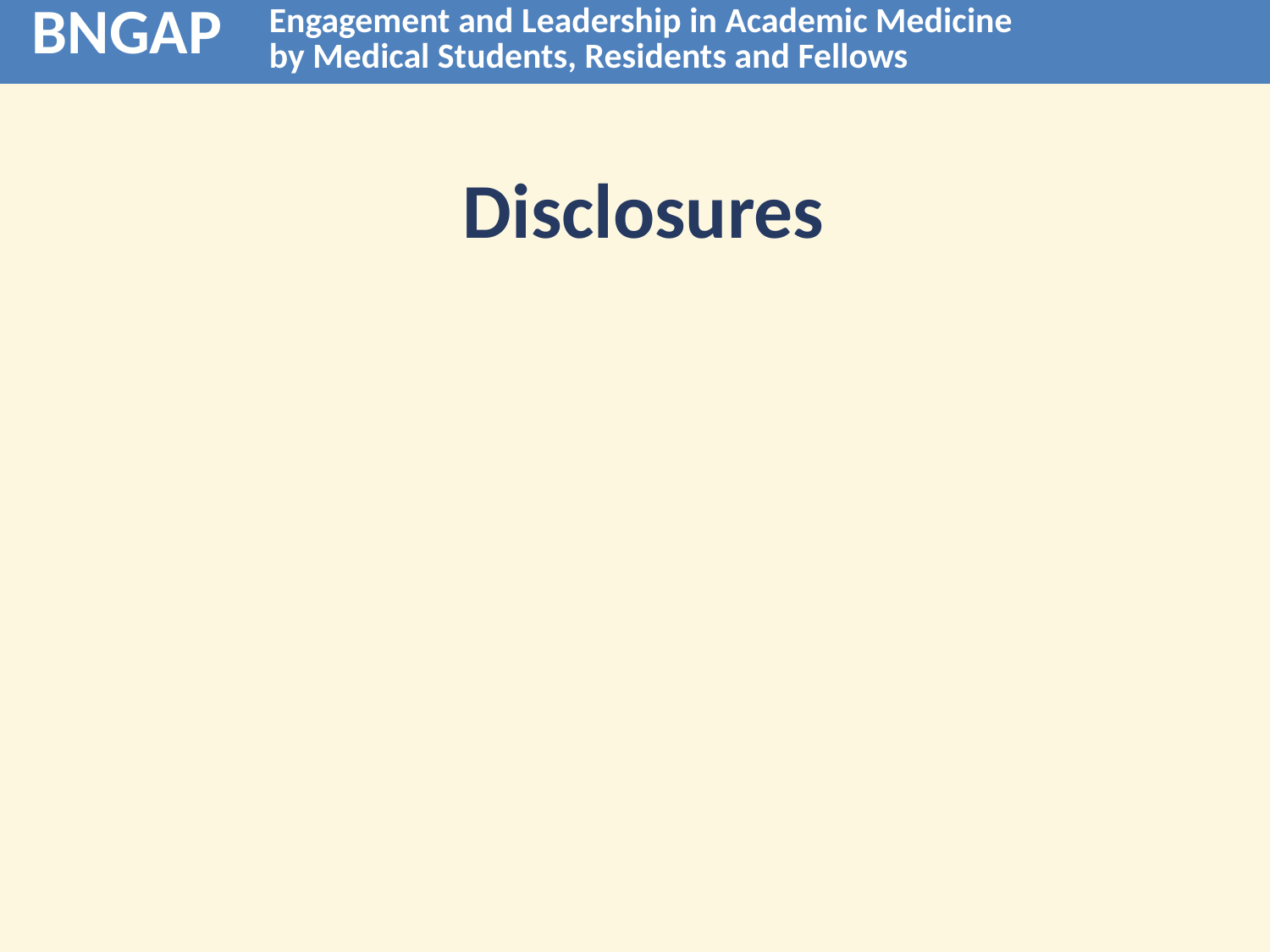

# Disclosures

## Slide 3
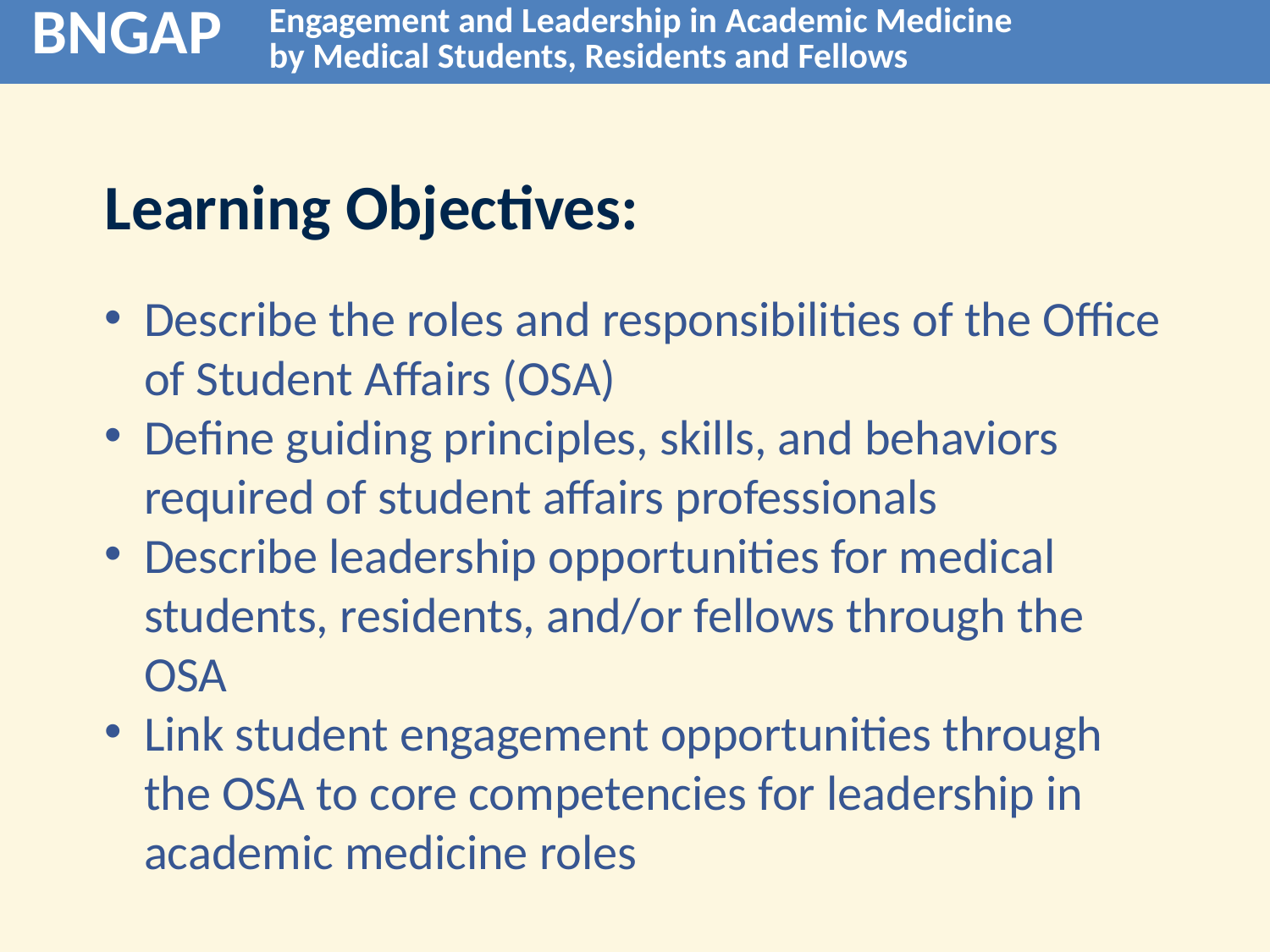

Learning Objectives:
Describe the roles and responsibilities of the Office of Student Affairs (OSA)
Define guiding principles, skills, and behaviors required of student affairs professionals
Describe leadership opportunities for medical students, residents, and/or fellows through the OSA
Link student engagement opportunities through the OSA to core competencies for leadership in academic medicine roles

## Slide 4
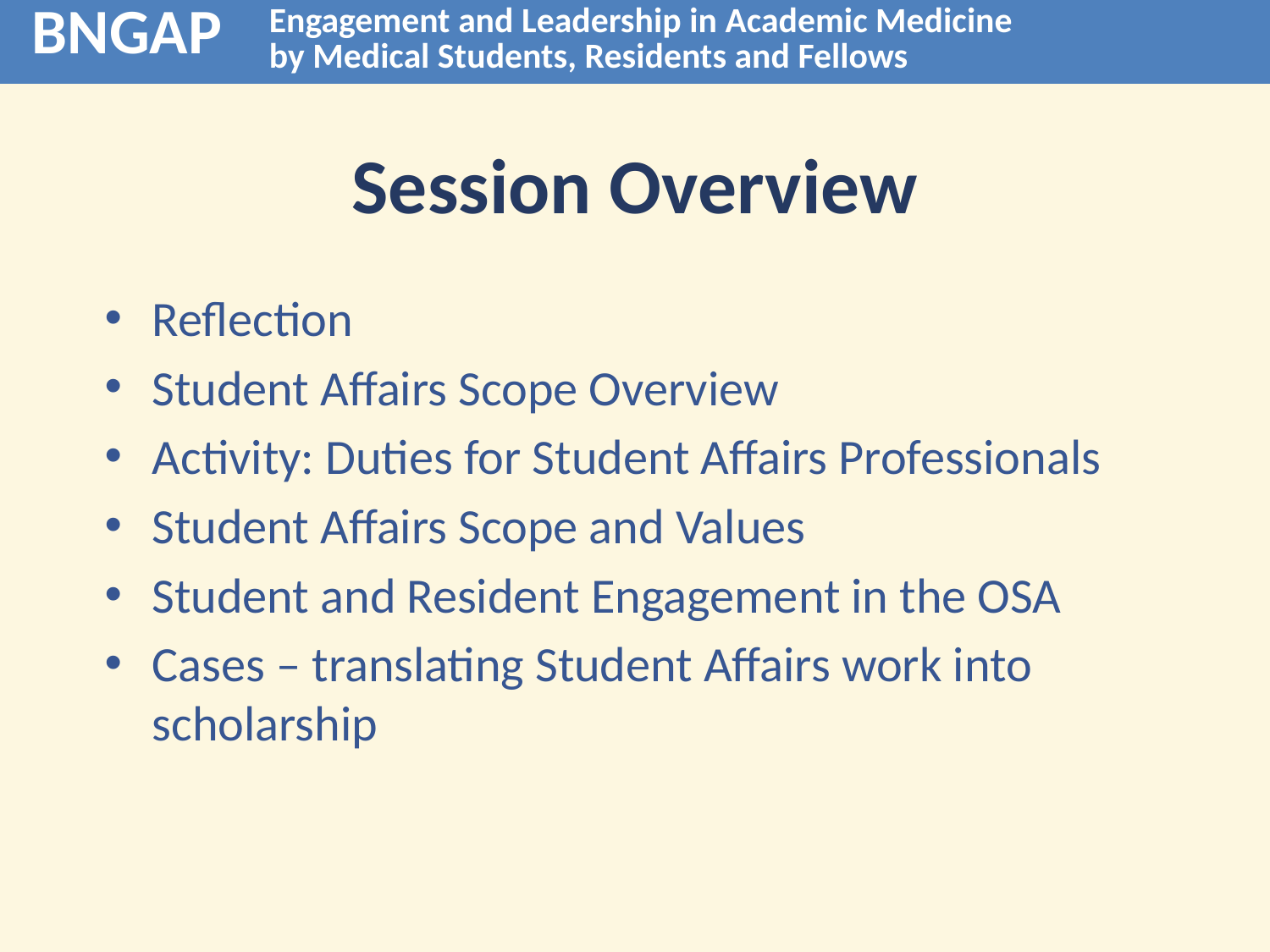

# Session Overview
Reflection
Student Affairs Scope Overview
Activity: Duties for Student Affairs Professionals
Student Affairs Scope and Values
Student and Resident Engagement in the OSA
Cases – translating Student Affairs work into scholarship

## Slide 5
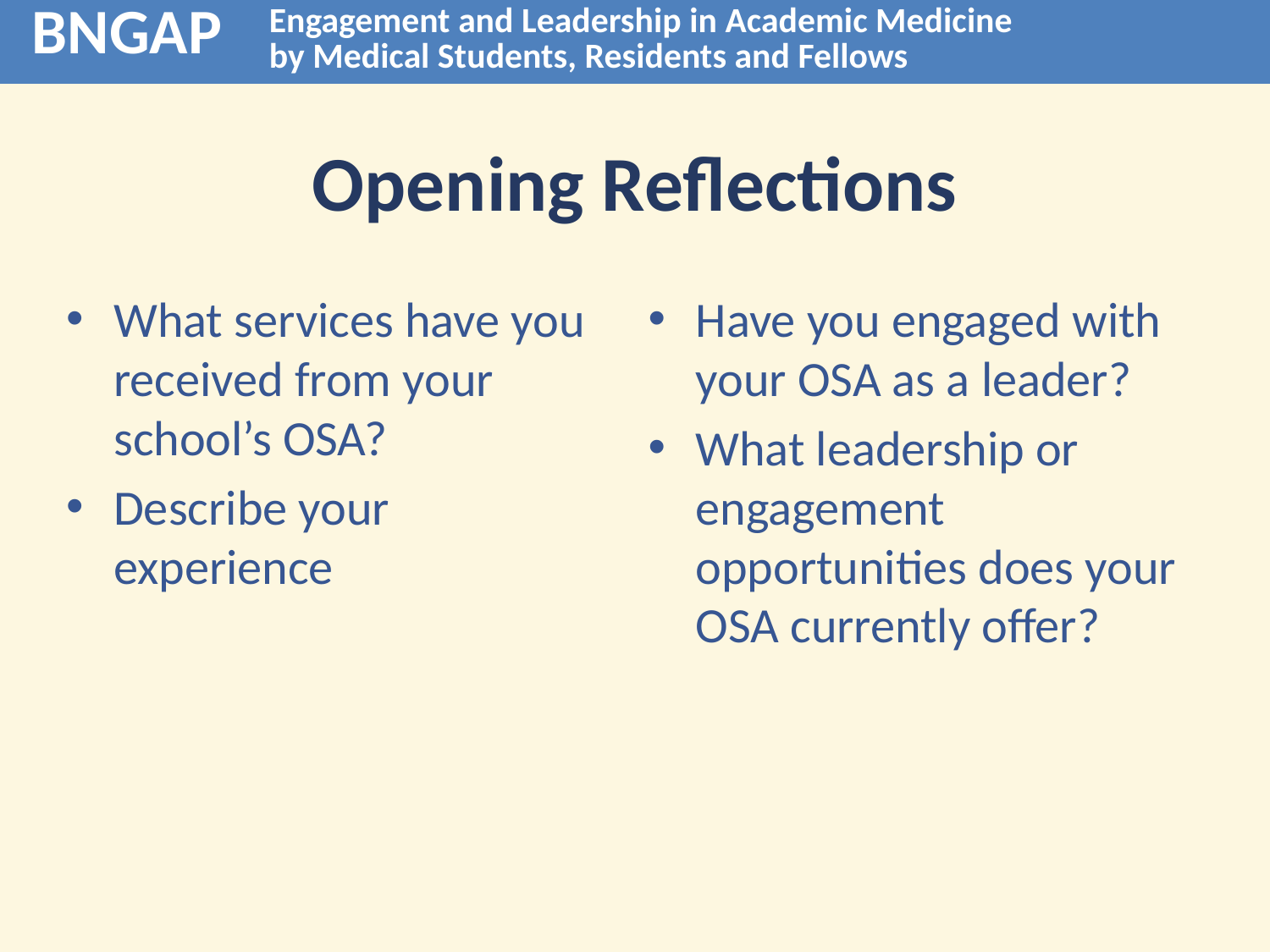

# Opening Reflections
What services have you received from your school’s OSA?
Describe your experience
Have you engaged with your OSA as a leader?
What leadership or engagement opportunities does your OSA currently offer?

## Slide 6
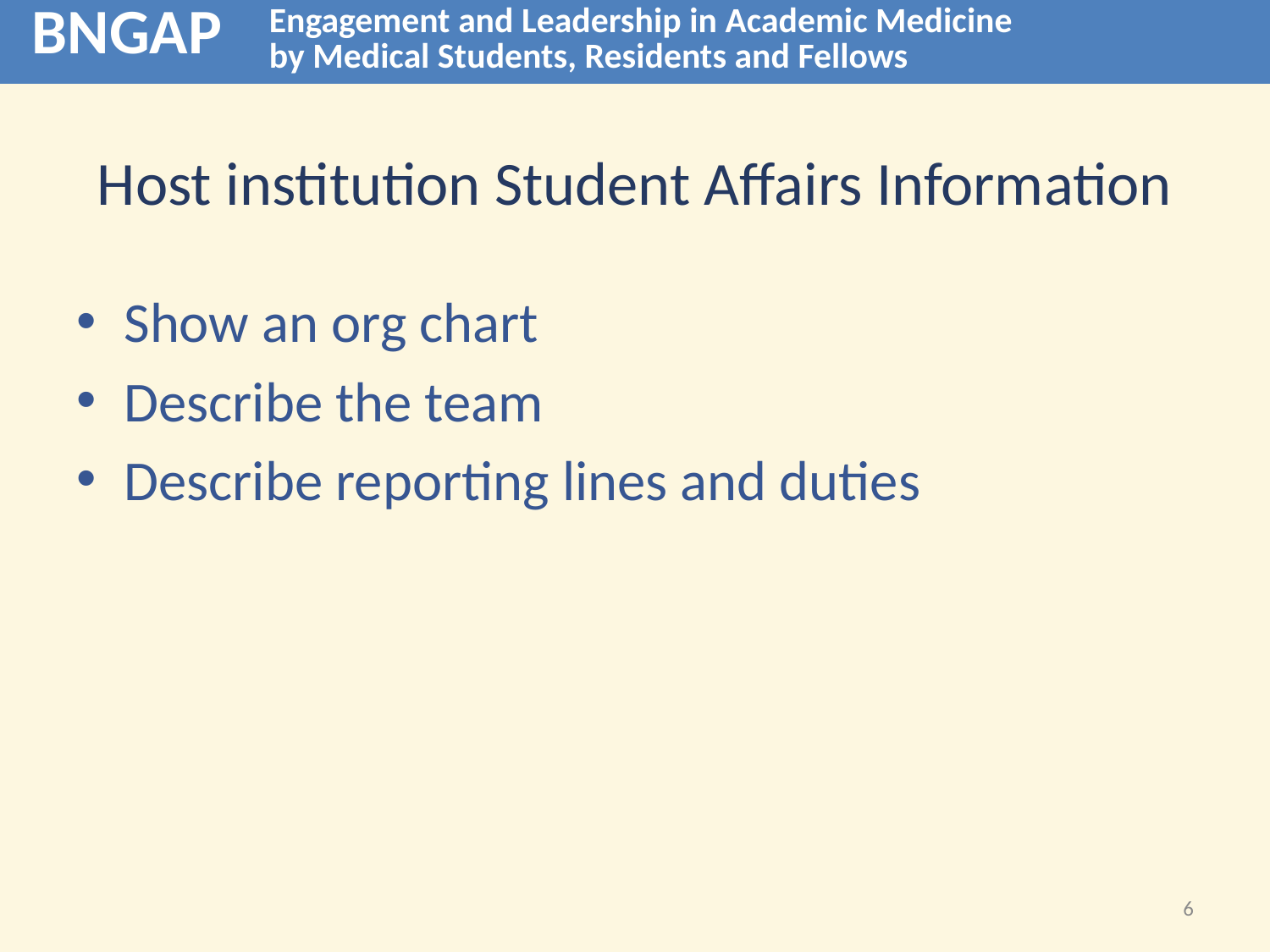

# Host institution Student Affairs Information
Show an org chart
Describe the team
Describe reporting lines and duties
6

## Slide 7
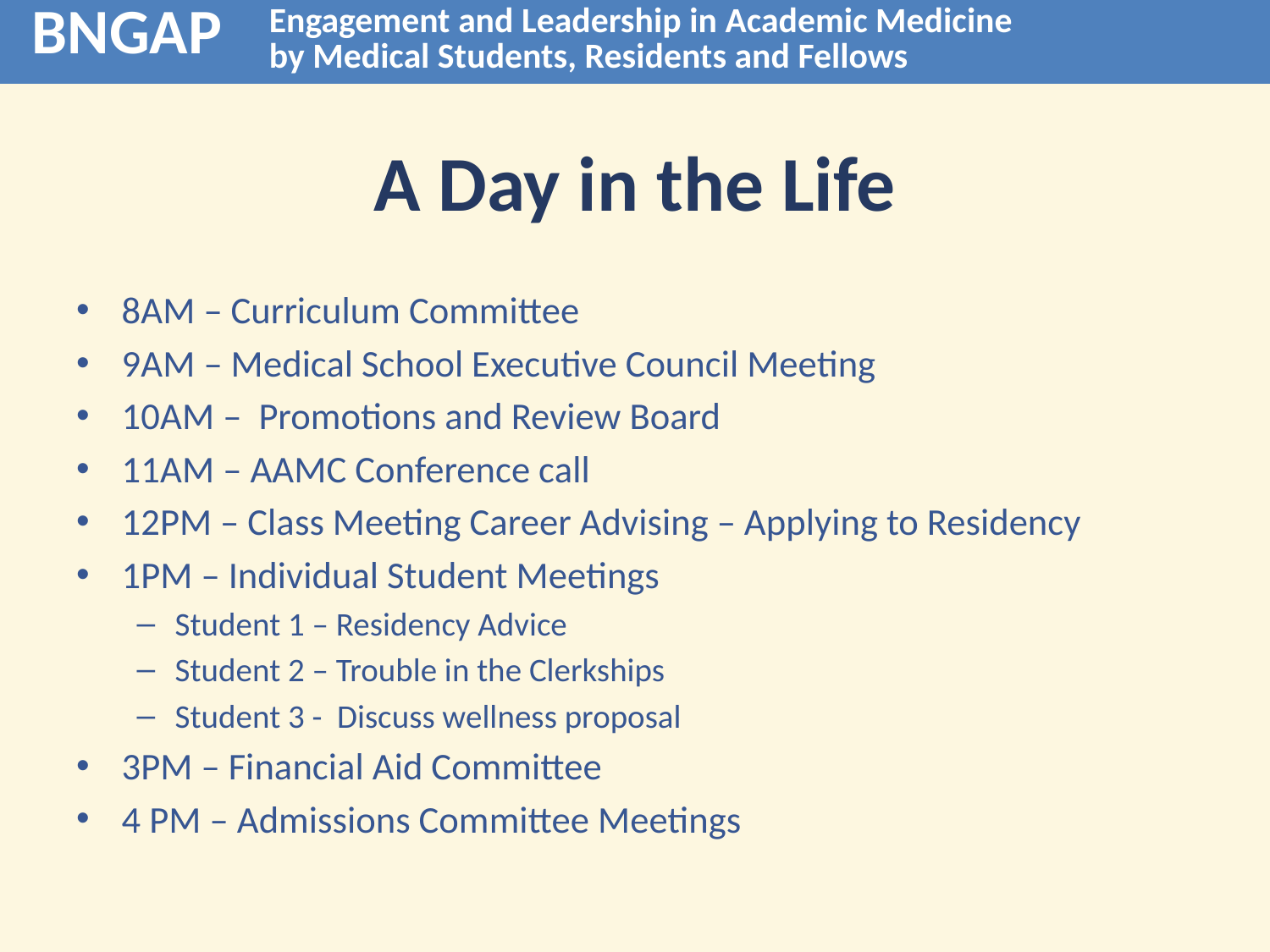

# A Day in the Life
8AM – Curriculum Committee
9AM – Medical School Executive Council Meeting
10AM – Promotions and Review Board
11AM – AAMC Conference call
12PM – Class Meeting Career Advising – Applying to Residency
1PM – Individual Student Meetings
Student 1 – Residency Advice
Student 2 – Trouble in the Clerkships
Student 3 - Discuss wellness proposal
3PM – Financial Aid Committee
4 PM – Admissions Committee Meetings

## Slide 8
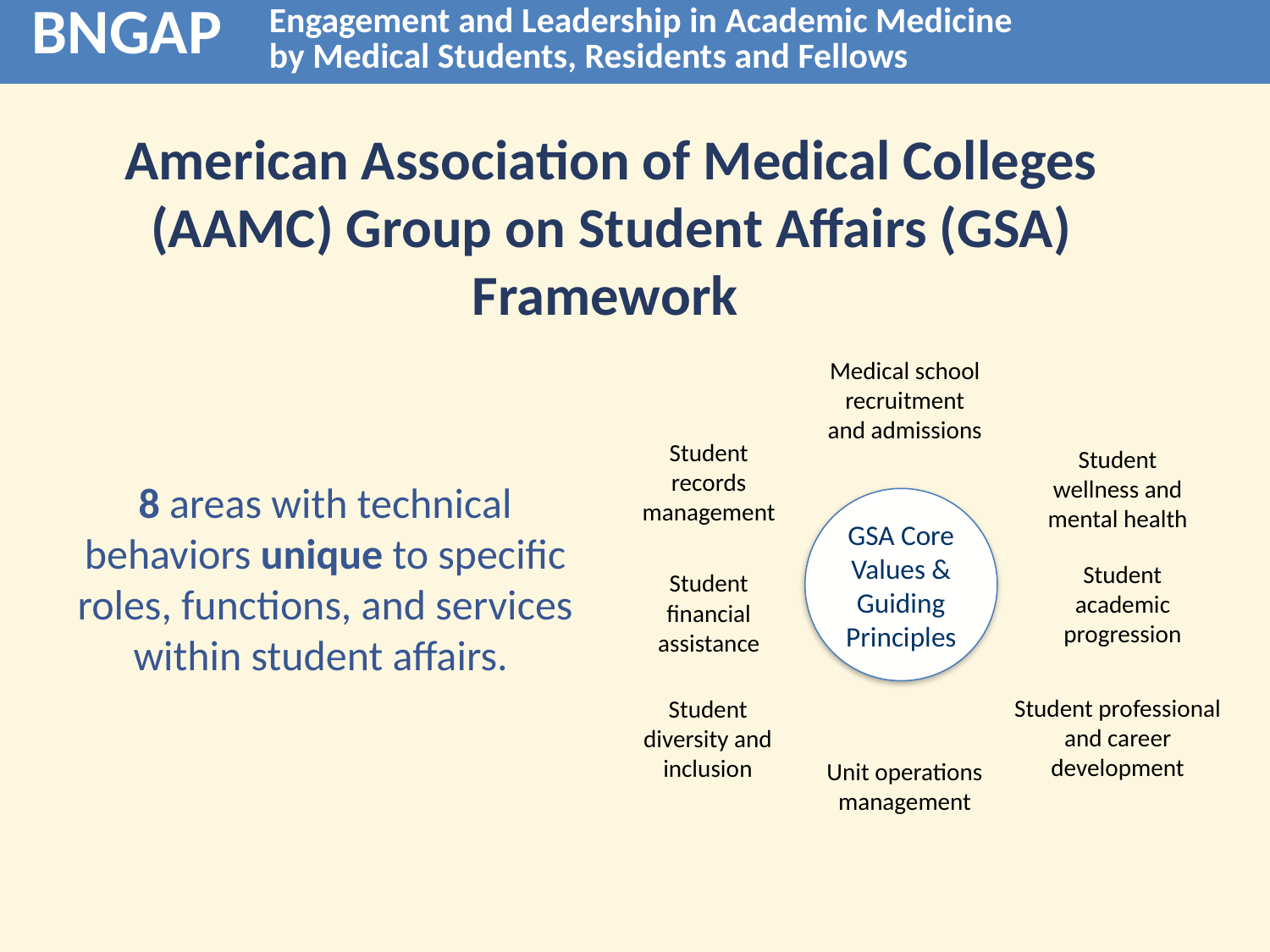

# American Association of Medical Colleges (AAMC) Group on Student Affairs (GSA) Framework
Medical school recruitment and admissions
Student records management
Student wellness and mental health
8 areas with technical behaviors unique to specific roles, functions, and services within student affairs.
GSA Core Values & Guiding Principles
Student academic progression
Student financial assistance
Student professional and career development
Student diversity and inclusion
Unit operations management

## Slide 9
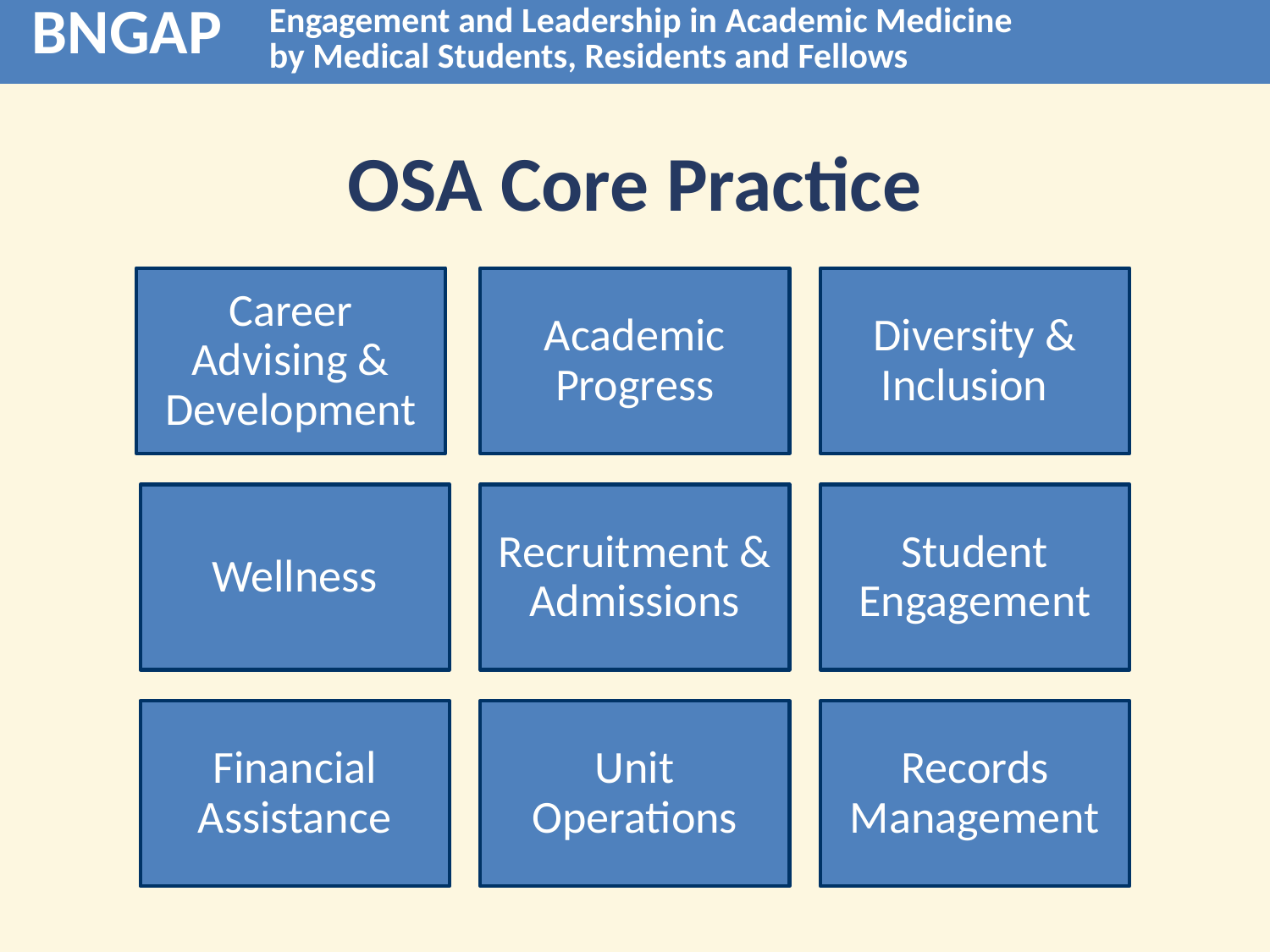

# OSA Core Practice

## Slide 10
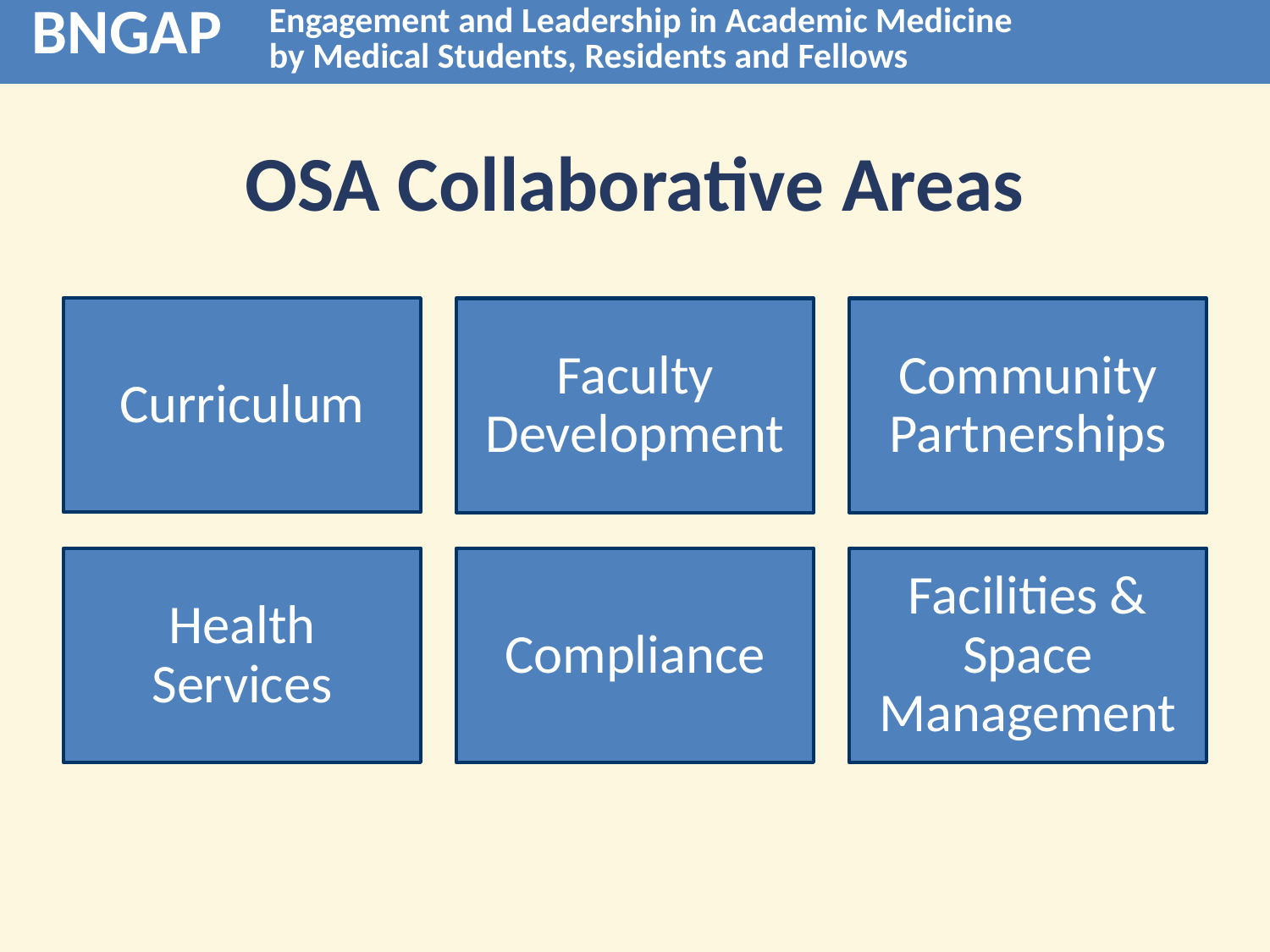

# OSA Collaborative Areas

## Slide 11
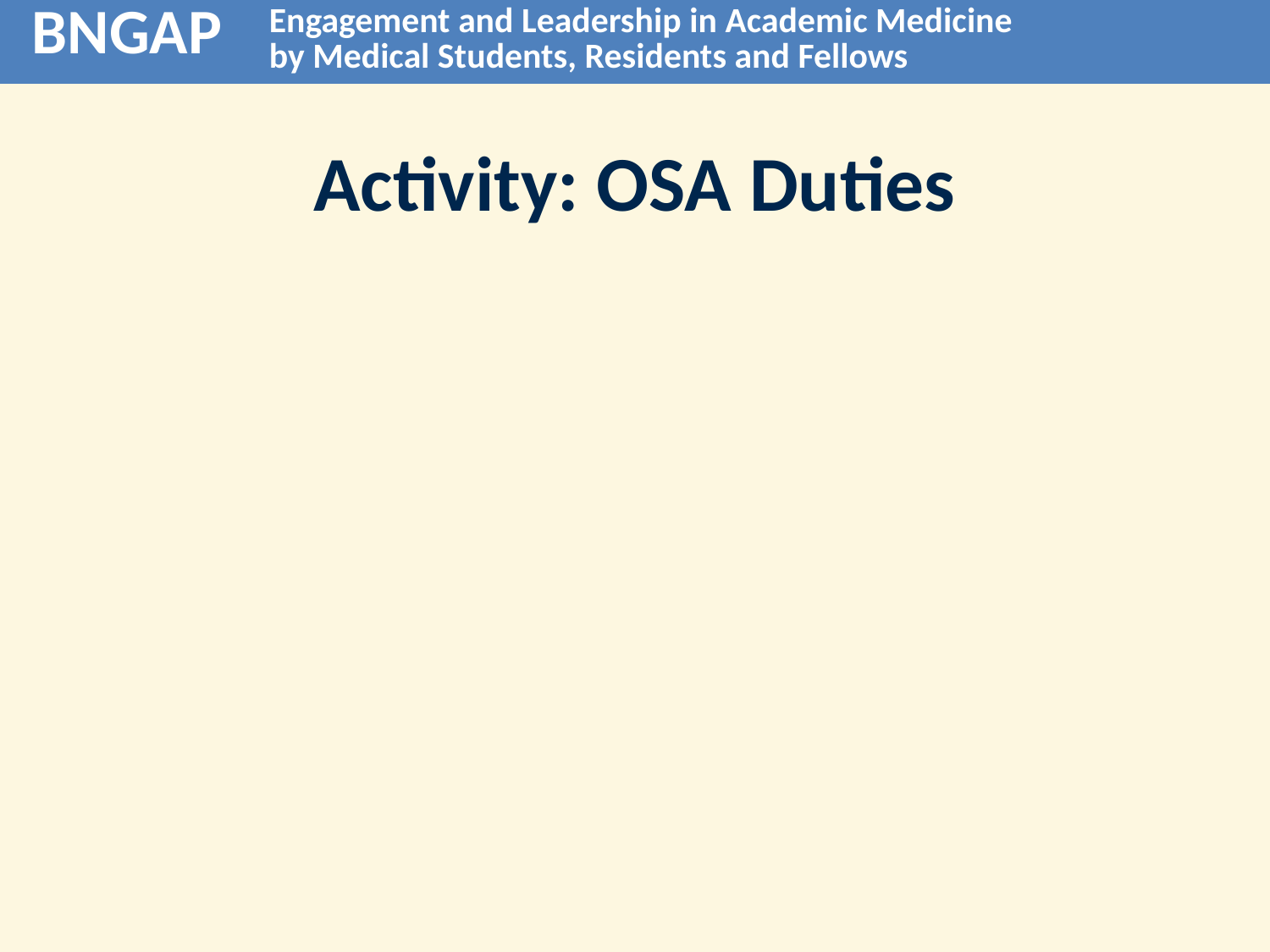

# Activity: OSA Duties

## Slide 12
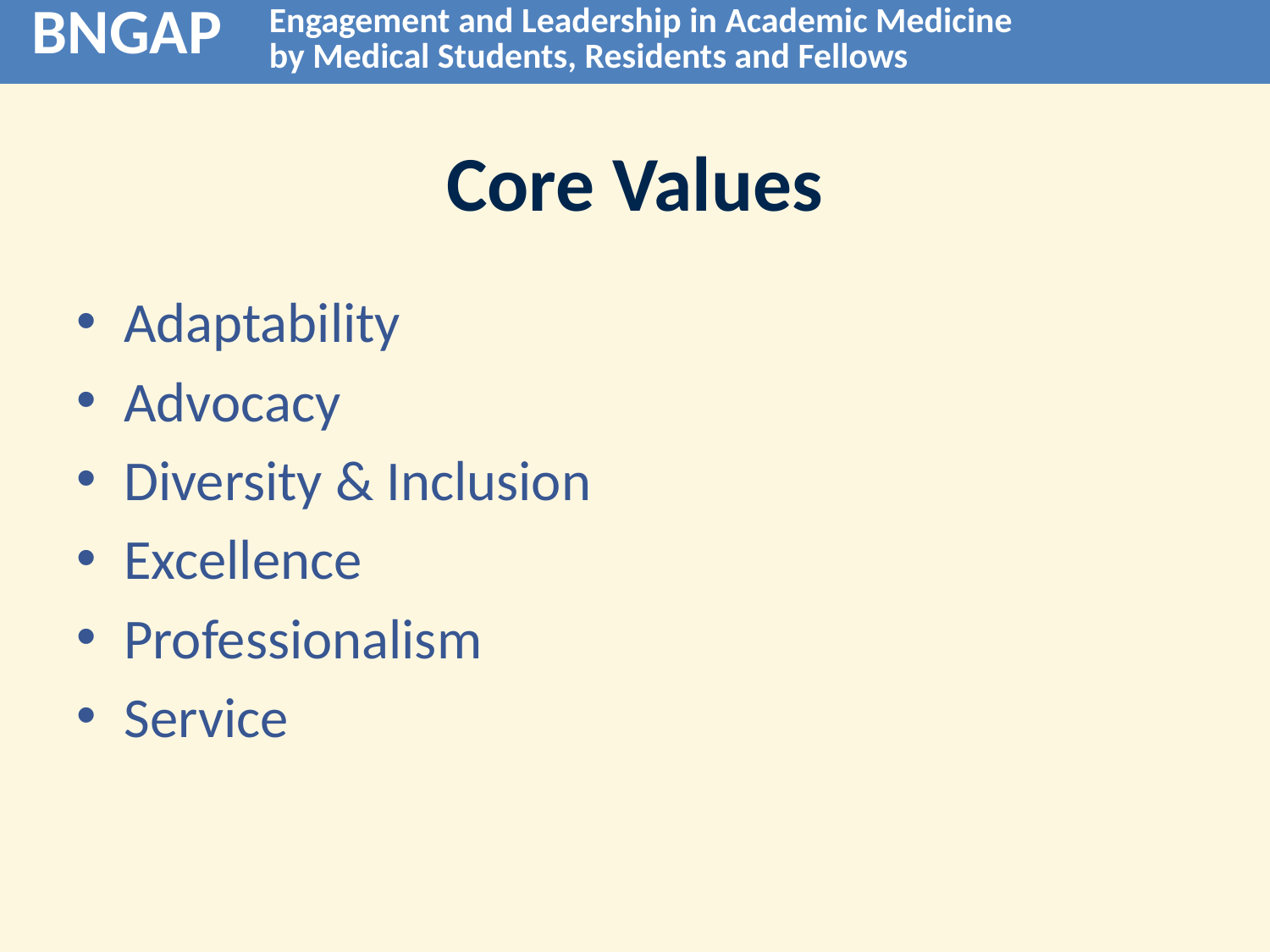

# Core Values
Adaptability
Advocacy
Diversity & Inclusion
Excellence
Professionalism
Service

## Slide 13
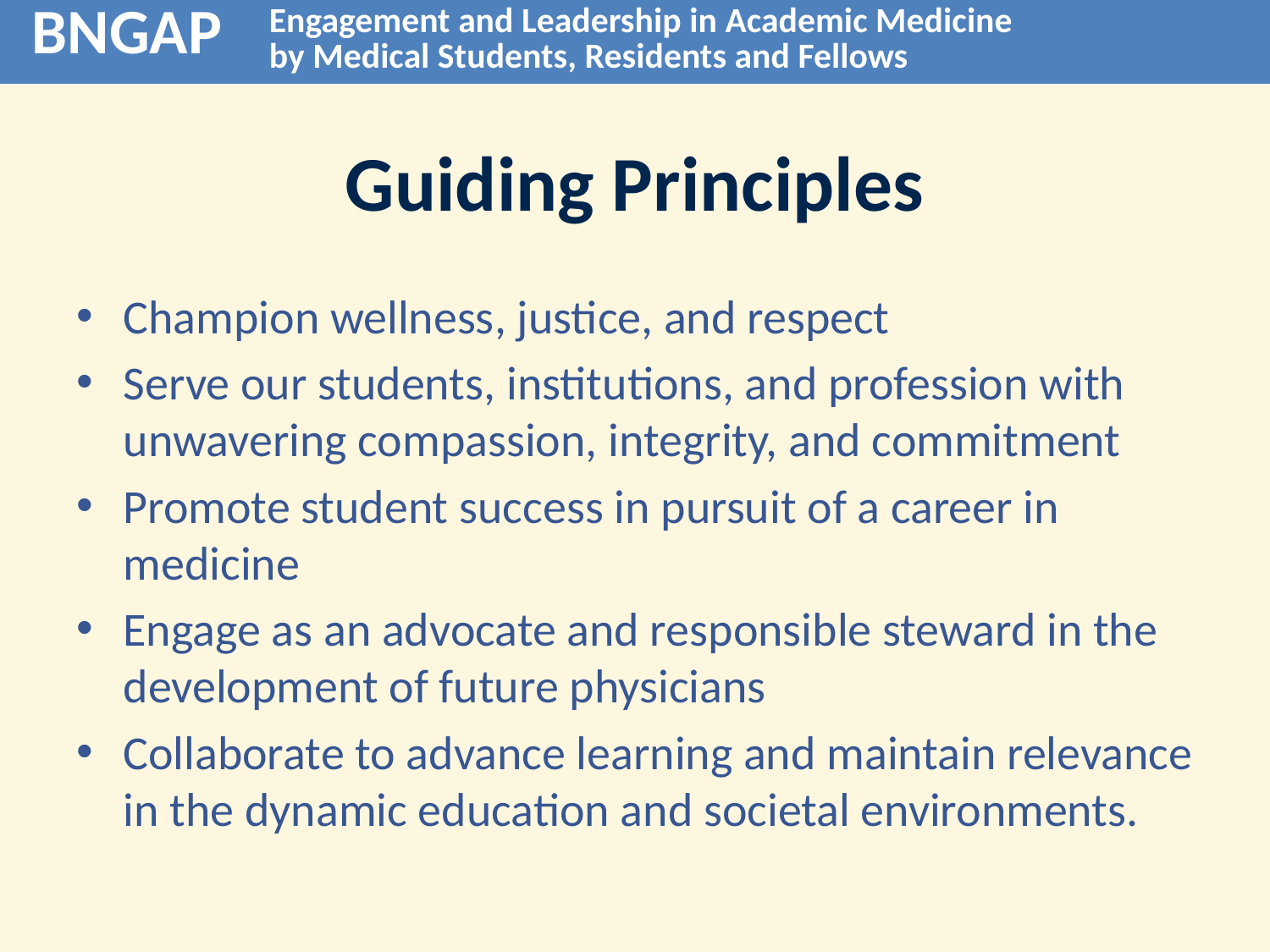

# Guiding Principles
Champion wellness, justice, and respect
Serve our students, institutions, and profession with unwavering compassion, integrity, and commitment
Promote student success in pursuit of a career in medicine
Engage as an advocate and responsible steward in the development of future physicians
Collaborate to advance learning and maintain relevance in the dynamic education and societal environments.

## Slide 14
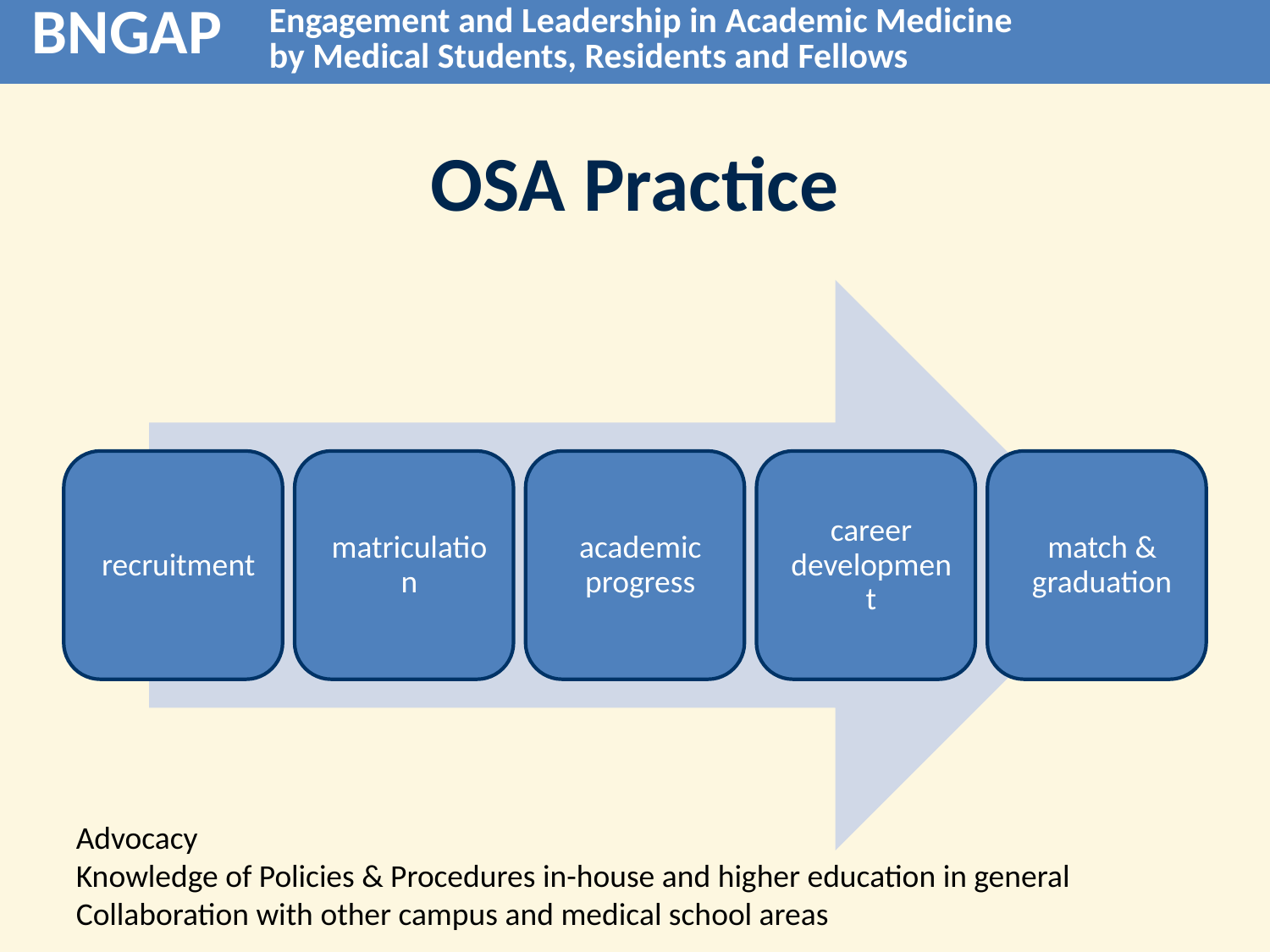

# OSA Practice
Advocacy
Knowledge of Policies & Procedures in-house and higher education in general
Collaboration with other campus and medical school areas

## Slide 15
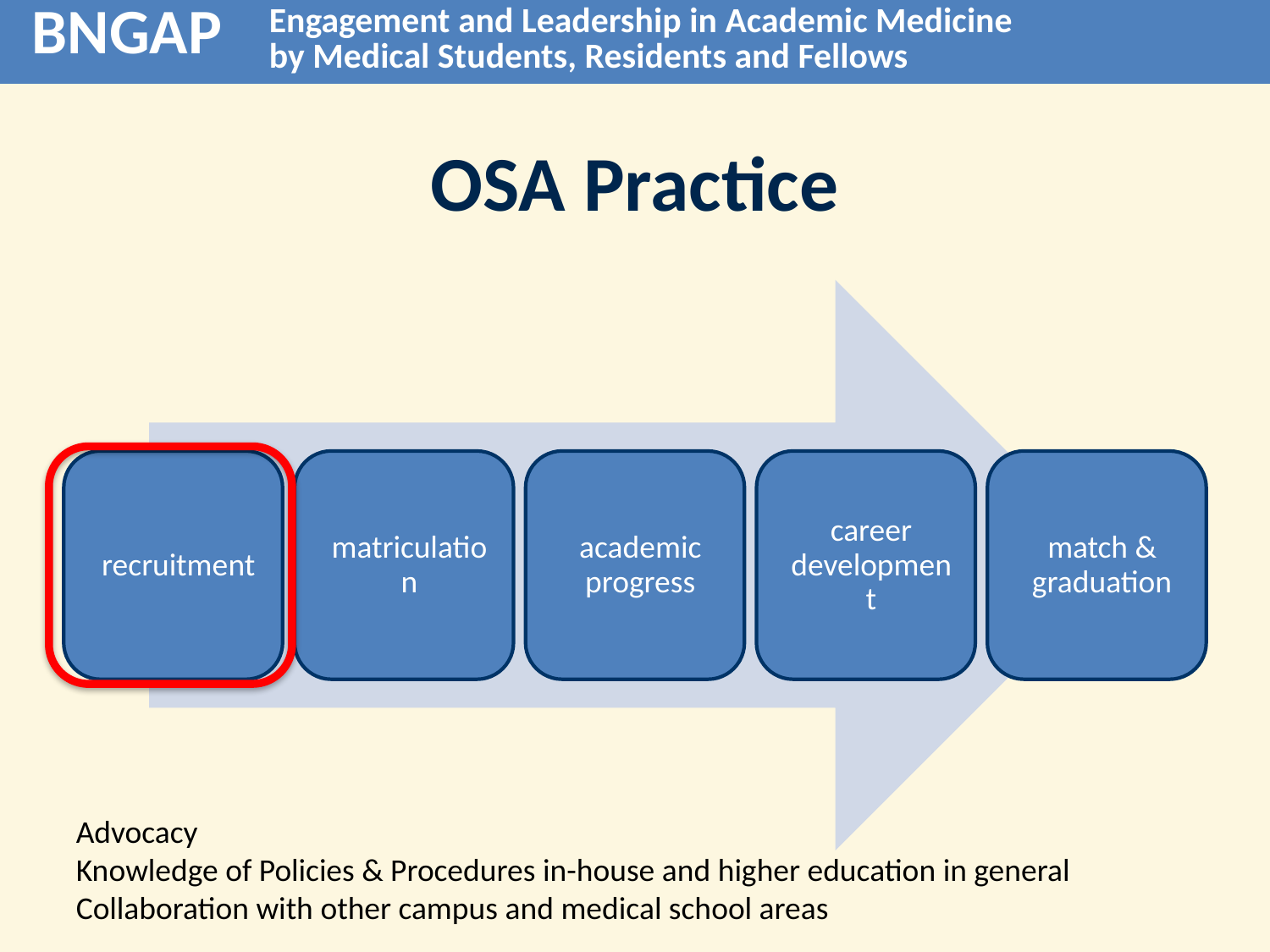

# OSA Practice
Advocacy
Knowledge of Policies & Procedures in-house and higher education in general
Collaboration with other campus and medical school areas

## Slide 16
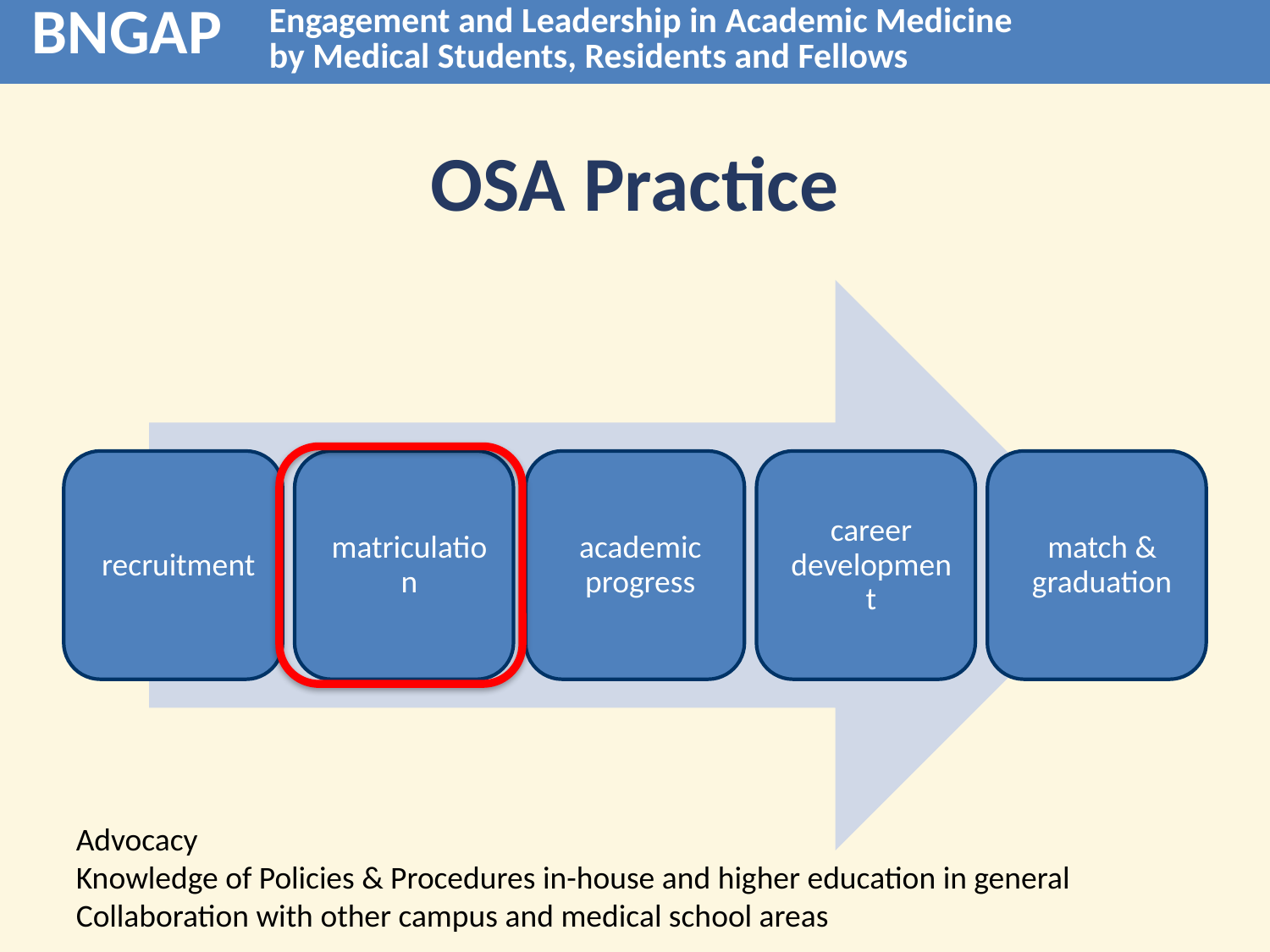

# OSA Practice
Advocacy
Knowledge of Policies & Procedures in-house and higher education in general
Collaboration with other campus and medical school areas

## Slide 17
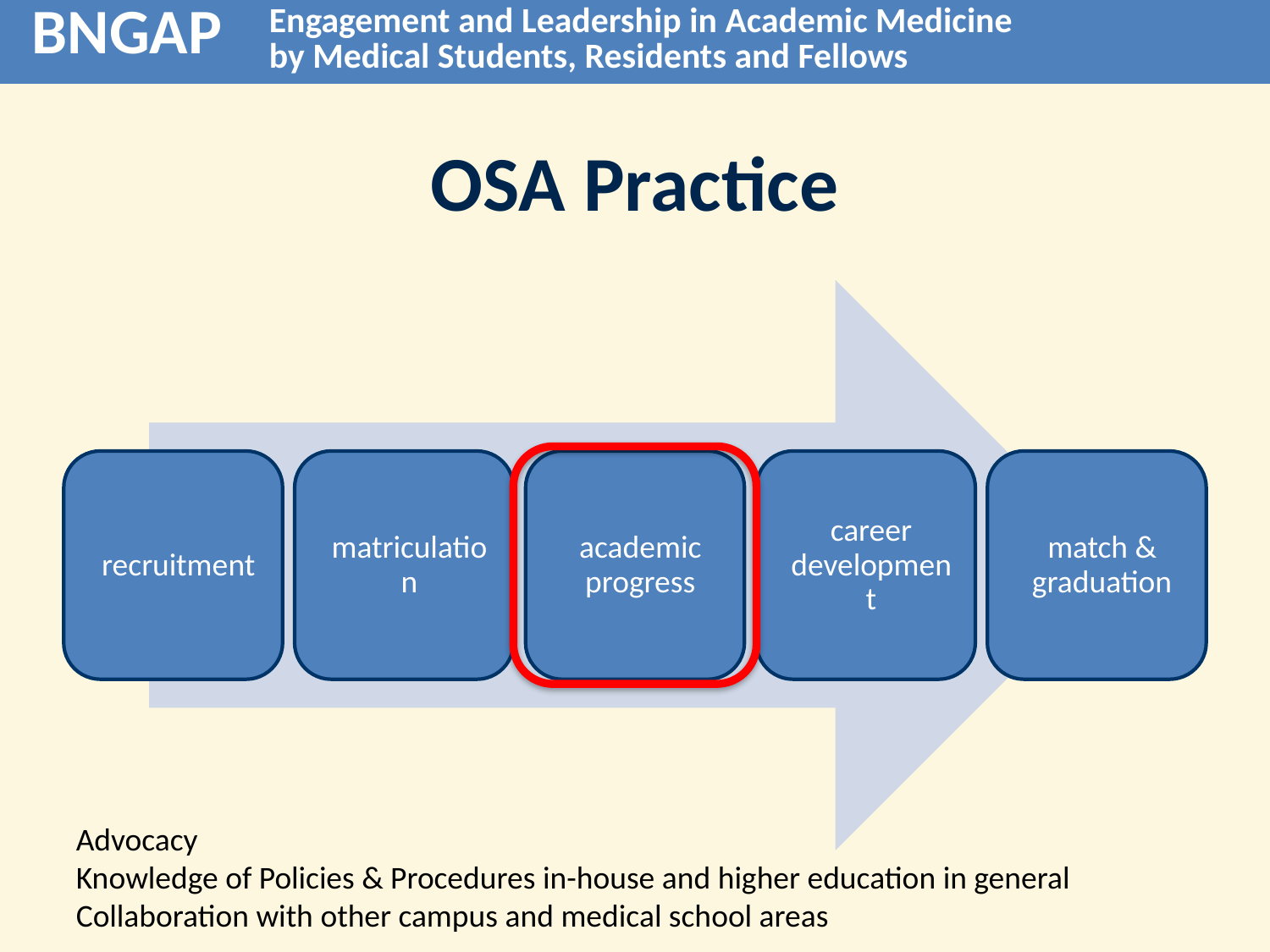

# OSA Practice
Advocacy
Knowledge of Policies & Procedures in-house and higher education in general
Collaboration with other campus and medical school areas

## Slide 18
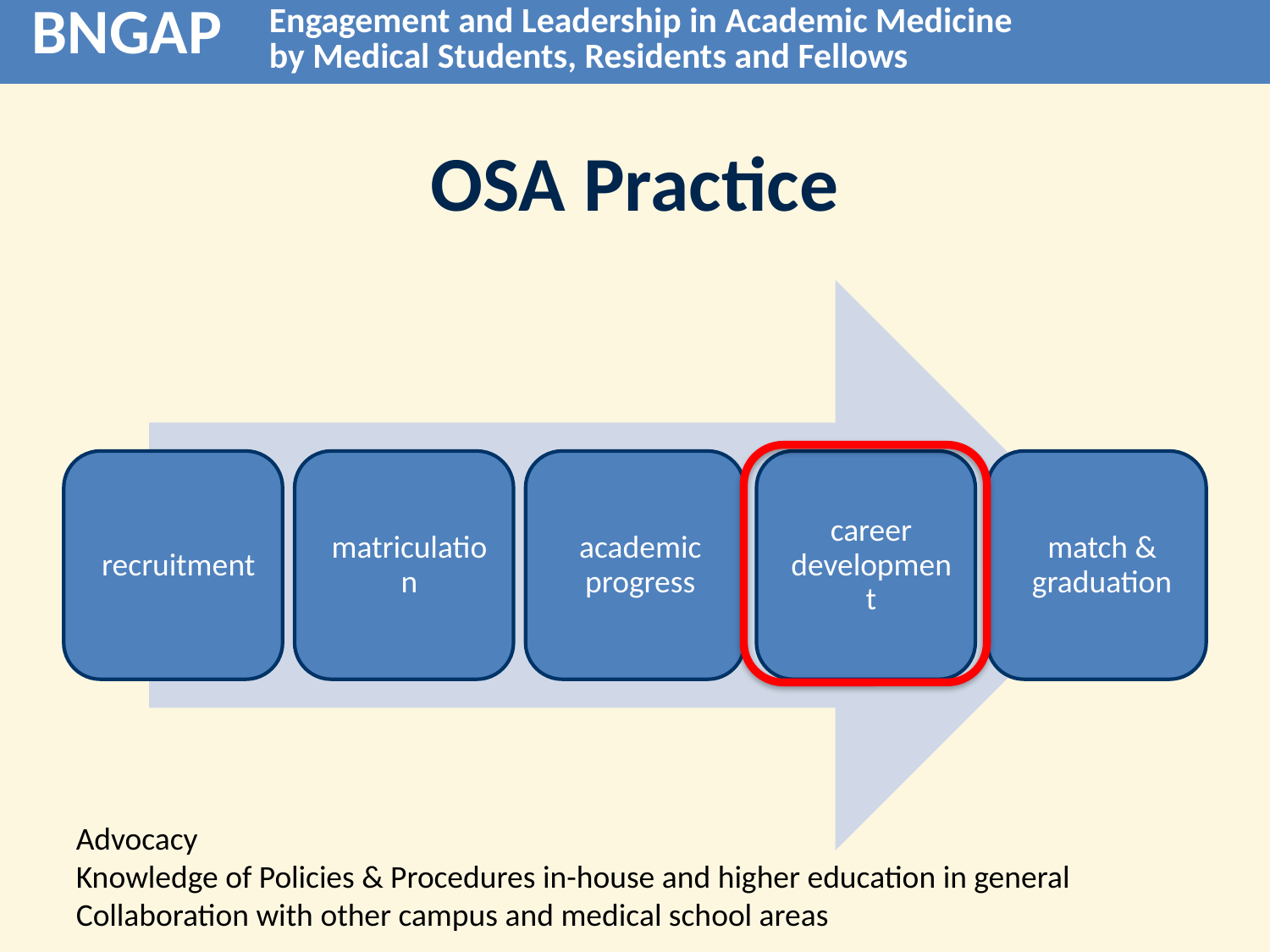

# OSA Practice
Advocacy
Knowledge of Policies & Procedures in-house and higher education in general
Collaboration with other campus and medical school areas

## Slide 19
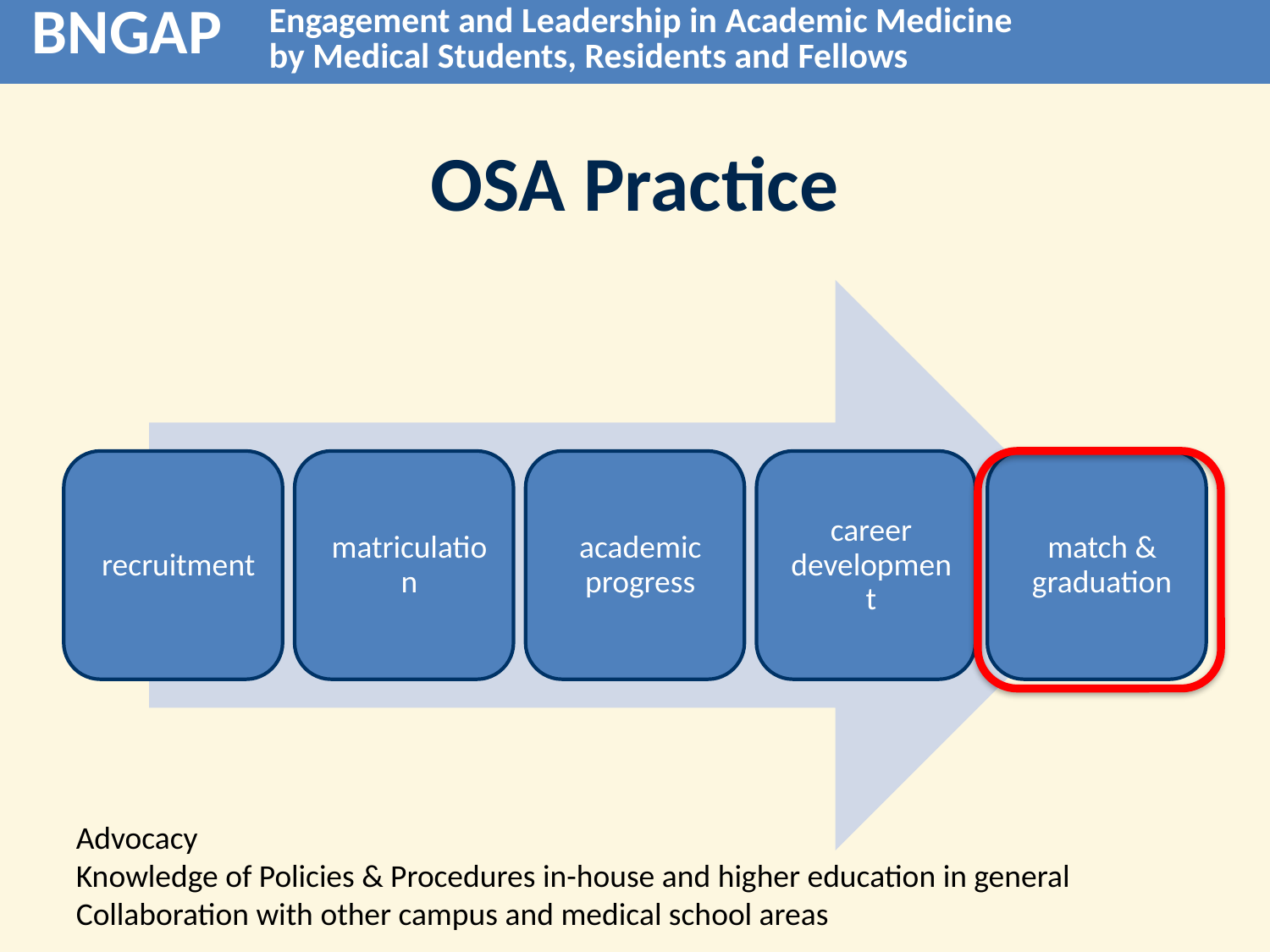

# OSA Practice
Advocacy
Knowledge of Policies & Procedures in-house and higher education in general
Collaboration with other campus and medical school areas

## Slide 20
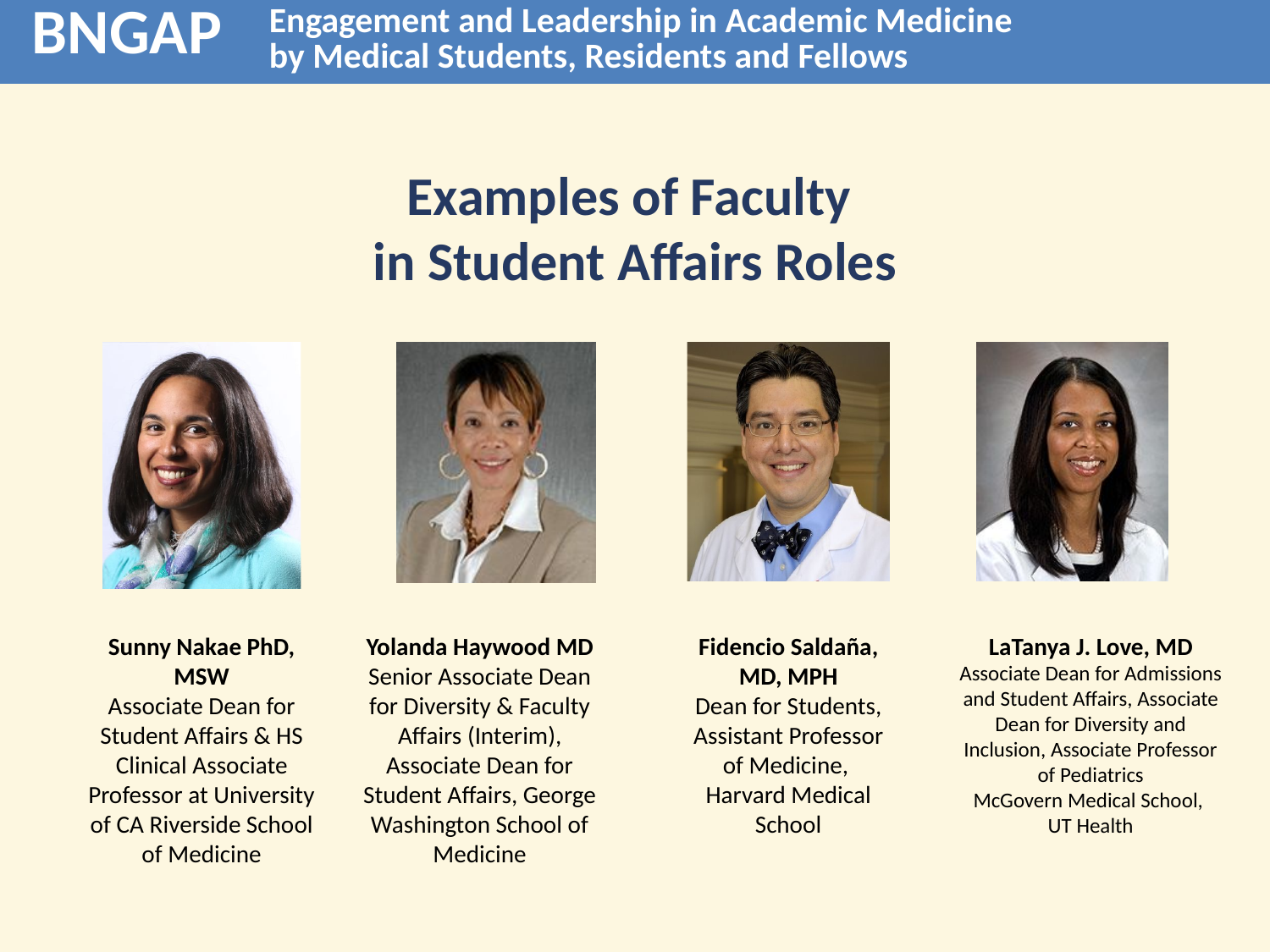

# Examples of Faculty in Student Affairs Roles
LaTanya J. Love, MD
Associate Dean for Admissions and Student Affairs, Associate Dean for Diversity and Inclusion, Associate Professor of Pediatrics
McGovern Medical School,
UT Health
Sunny Nakae PhD, MSW
Associate Dean for Student Affairs & HS Clinical Associate Professor at University of CA Riverside School of Medicine
Yolanda Haywood MD
Senior Associate Dean for Diversity & Faculty Affairs (Interim), Associate Dean for Student Affairs, George Washington School of Medicine
Fidencio Saldaña, MD, MPH
Dean for Students, Assistant Professor of Medicine,
Harvard Medical School

## Slide 21
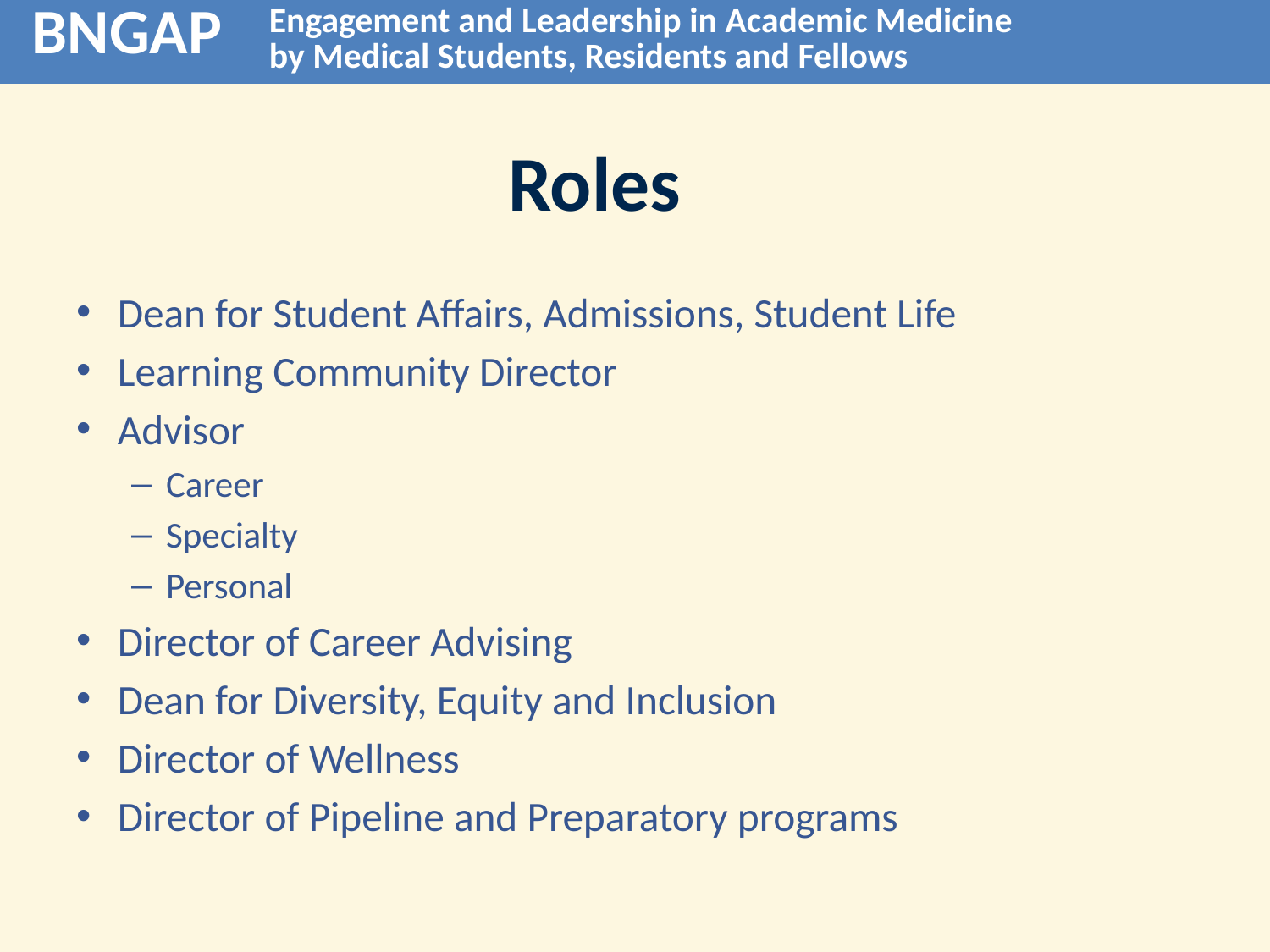

# Roles
Dean for Student Affairs, Admissions, Student Life
Learning Community Director
Advisor
Career
Specialty
Personal
Director of Career Advising
Dean for Diversity, Equity and Inclusion
Director of Wellness
Director of Pipeline and Preparatory programs

## Slide 22
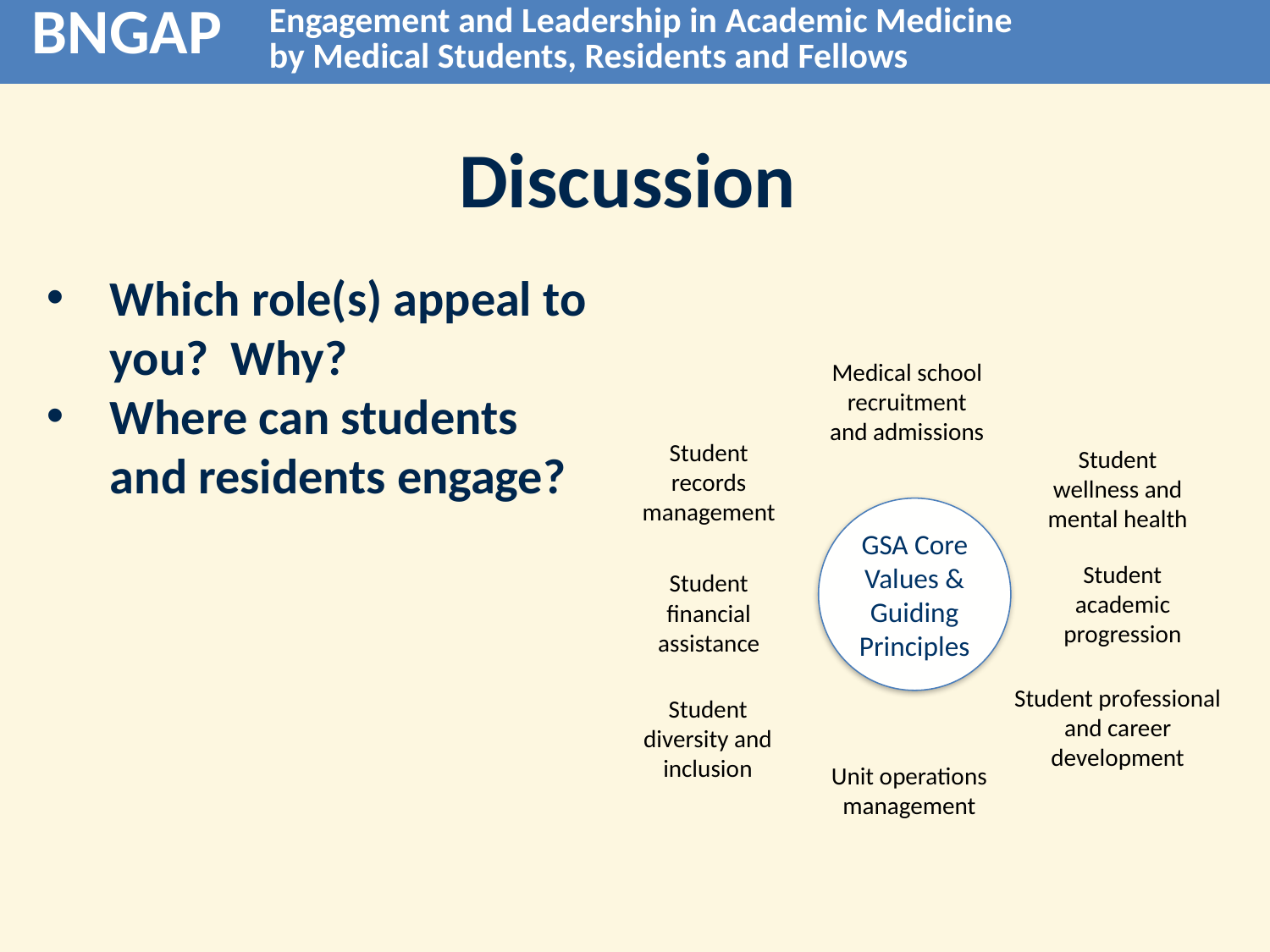

# Discussion
Which role(s) appeal to you? Why?
Where can students and residents engage?
Medical school recruitment and admissions
Student records management
Student wellness and mental health
GSA Core Values & Guiding Principles
Student academic progression
Student financial assistance
Student professional and career development
Student diversity and inclusion
Unit operations management

## Slide 23
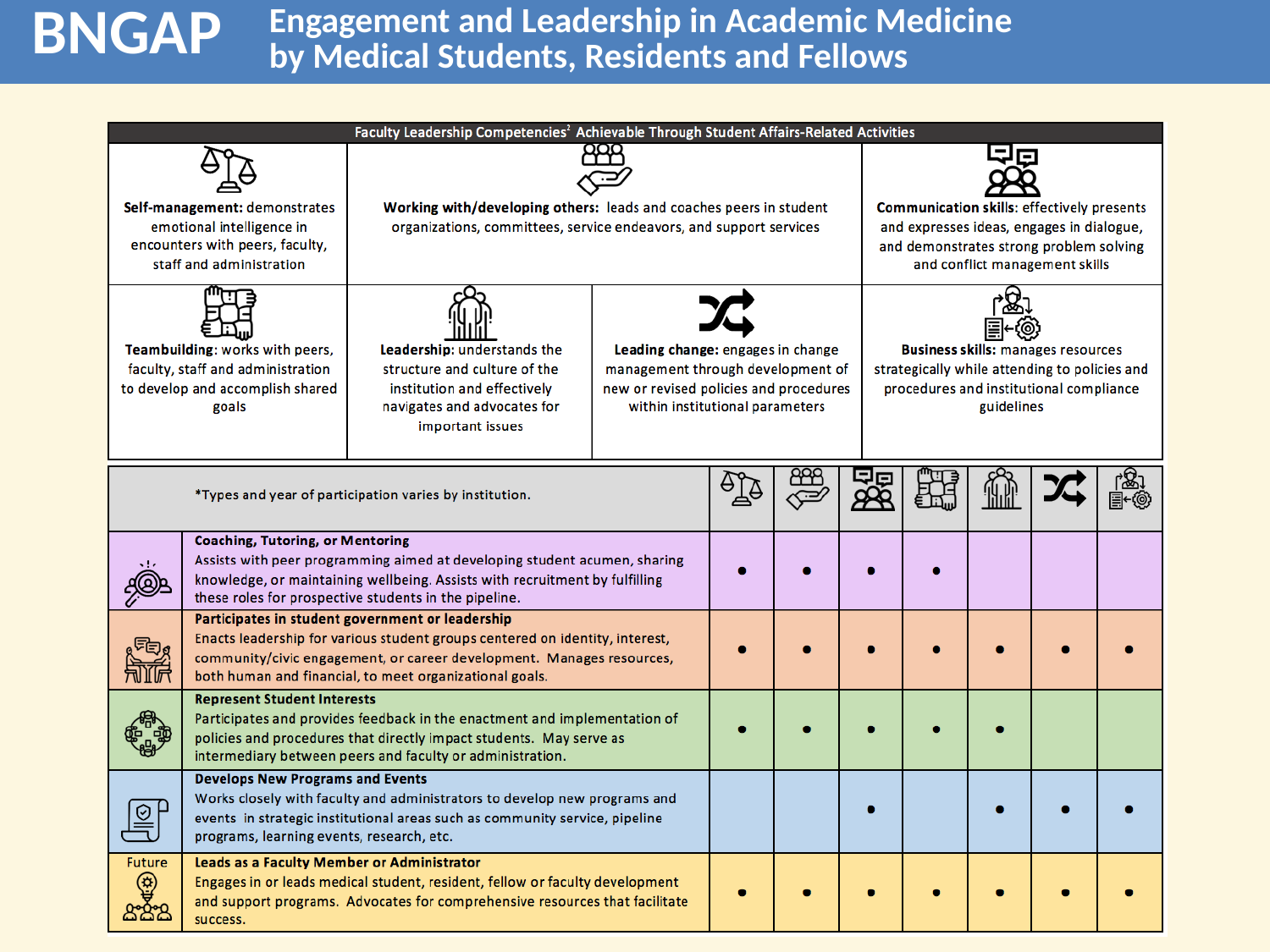

## Slide 24
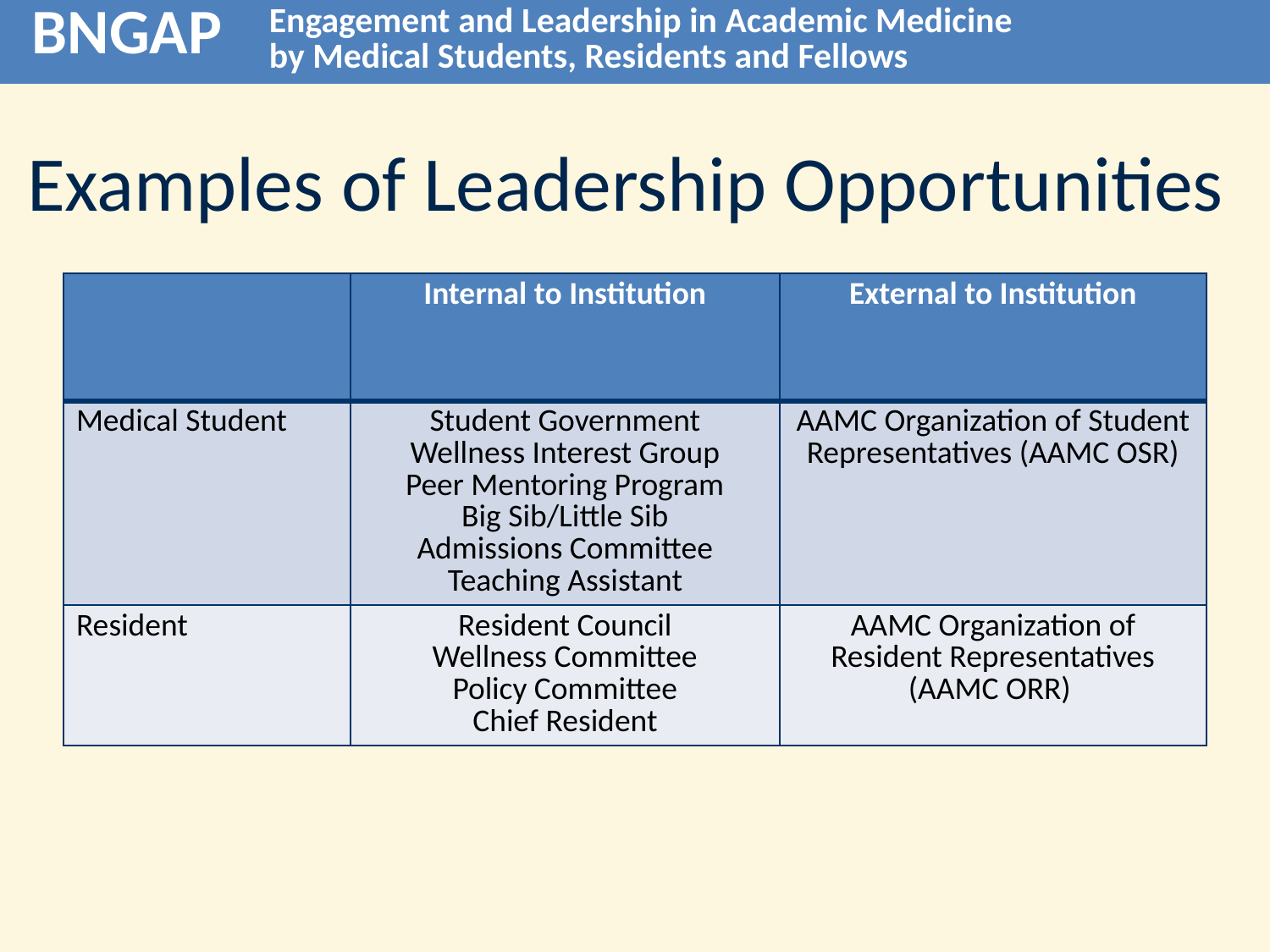

Examples of Leadership Opportunities
| | Internal to Institution | External to Institution |
| --- | --- | --- |
| Medical Student | Student Government Wellness Interest Group Peer Mentoring Program Big Sib/Little Sib Admissions Committee Teaching Assistant | AAMC Organization of Student Representatives (AAMC OSR) |
| Resident | Resident Council Wellness Committee Policy Committee Chief Resident | AAMC Organization of Resident Representatives (AAMC ORR) |

## Slide 25
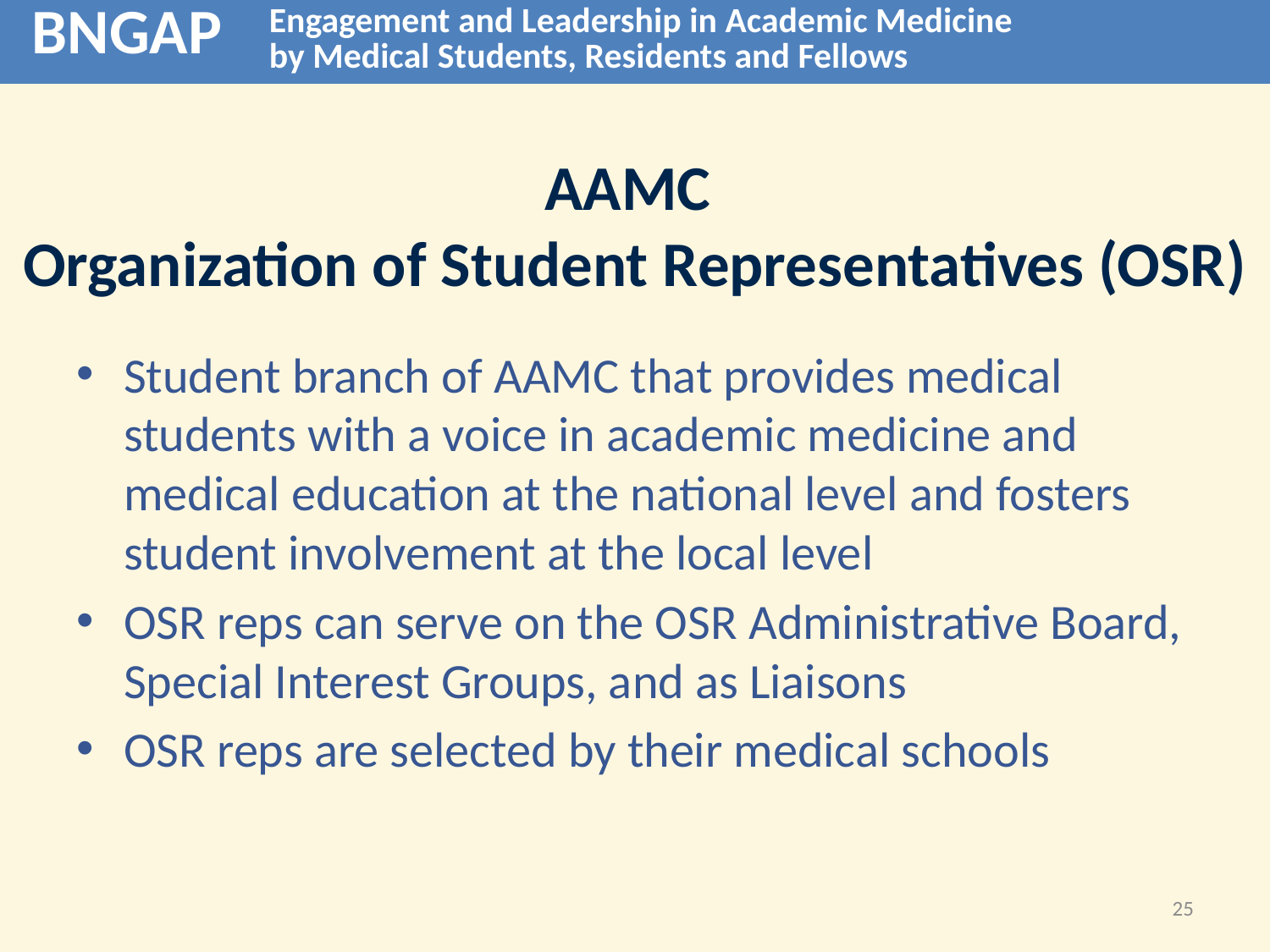

AAMC
Organization of Student Representatives (OSR)
Student branch of AAMC that provides medical students with a voice in academic medicine and medical education at the national level and fosters student involvement at the local level
OSR reps can serve on the OSR Administrative Board, Special Interest Groups, and as Liaisons
OSR reps are selected by their medical schools
25

## Slide 26
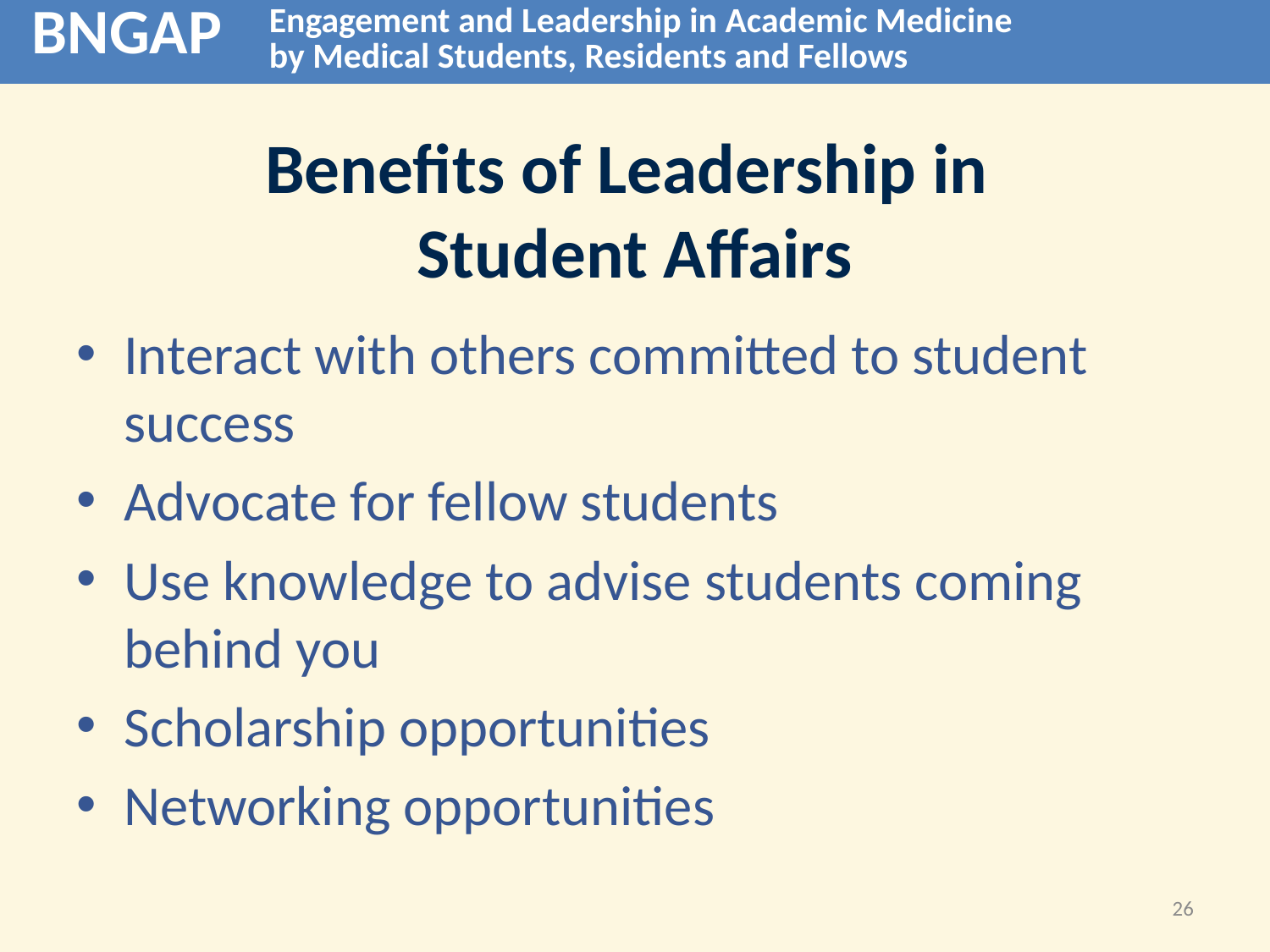

# Benefits of Leadership in Student Affairs
Interact with others committed to student success
Advocate for fellow students
Use knowledge to advise students coming behind you
Scholarship opportunities
Networking opportunities
26

## Slide 27
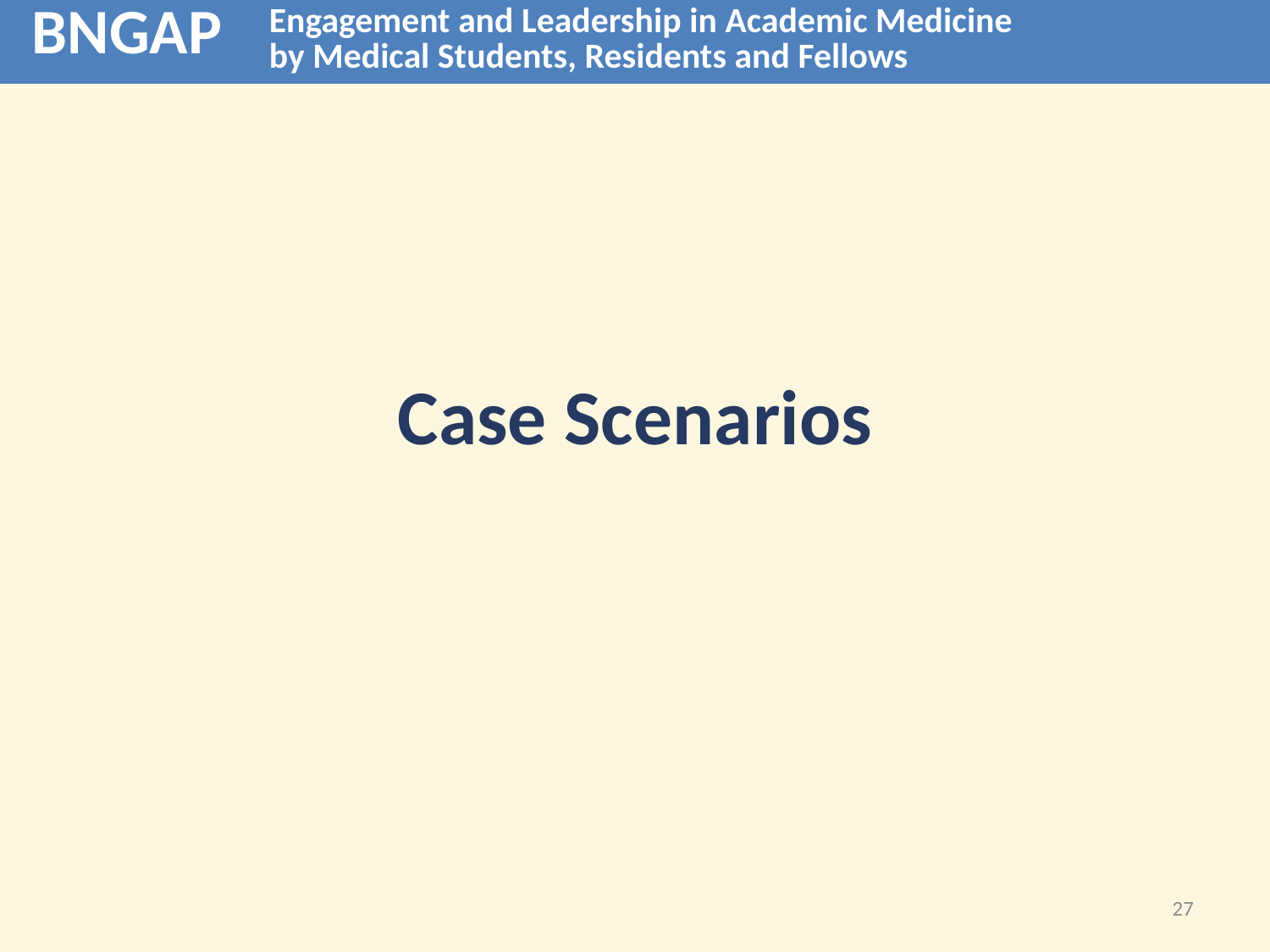

# Case Scenarios
27

## Slide 28
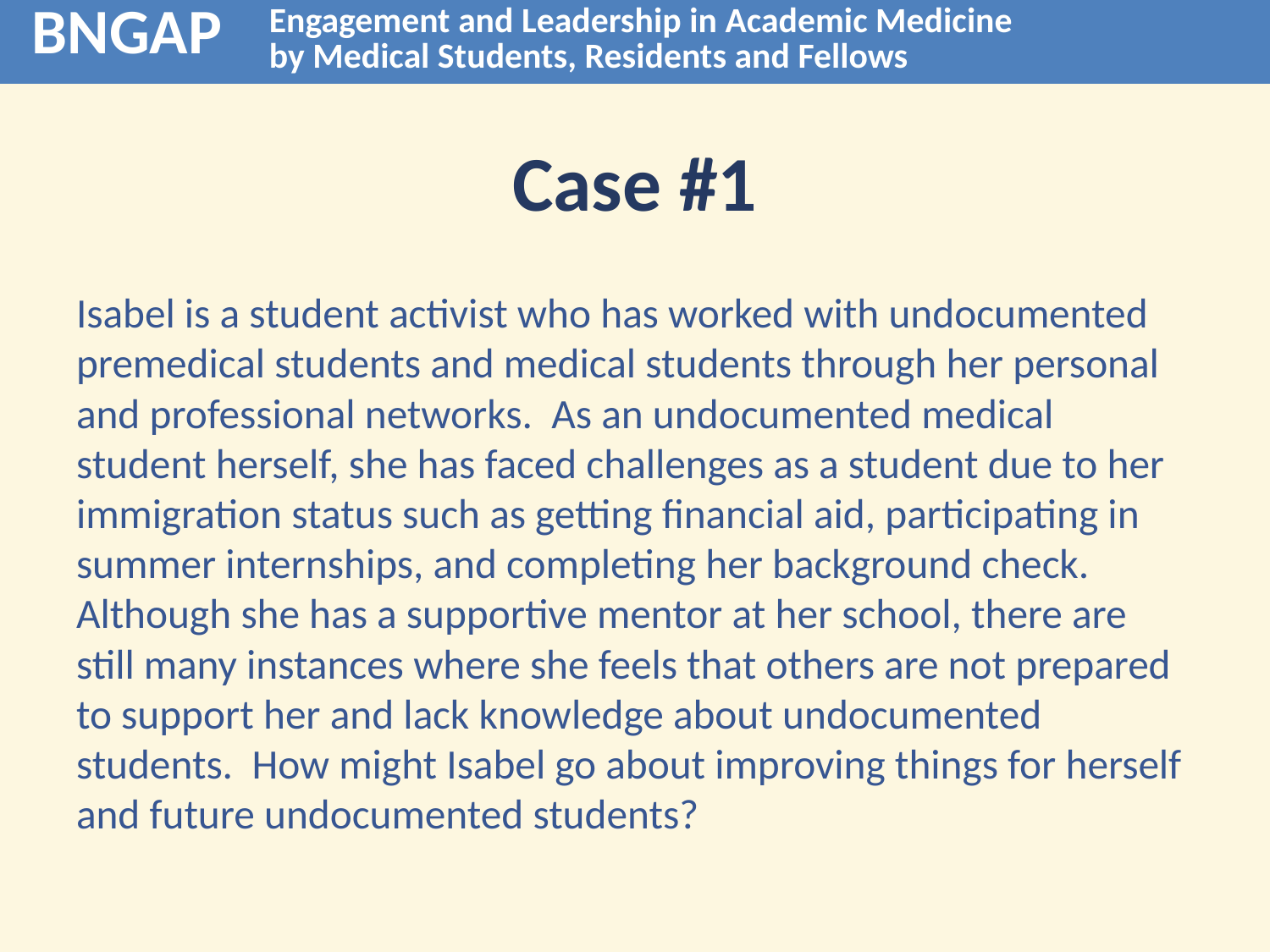

# Case #1
Isabel is a student activist who has worked with undocumented premedical students and medical students through her personal and professional networks. As an undocumented medical student herself, she has faced challenges as a student due to her immigration status such as getting financial aid, participating in summer internships, and completing her background check. Although she has a supportive mentor at her school, there are still many instances where she feels that others are not prepared to support her and lack knowledge about undocumented students. How might Isabel go about improving things for herself and future undocumented students?

## Slide 29
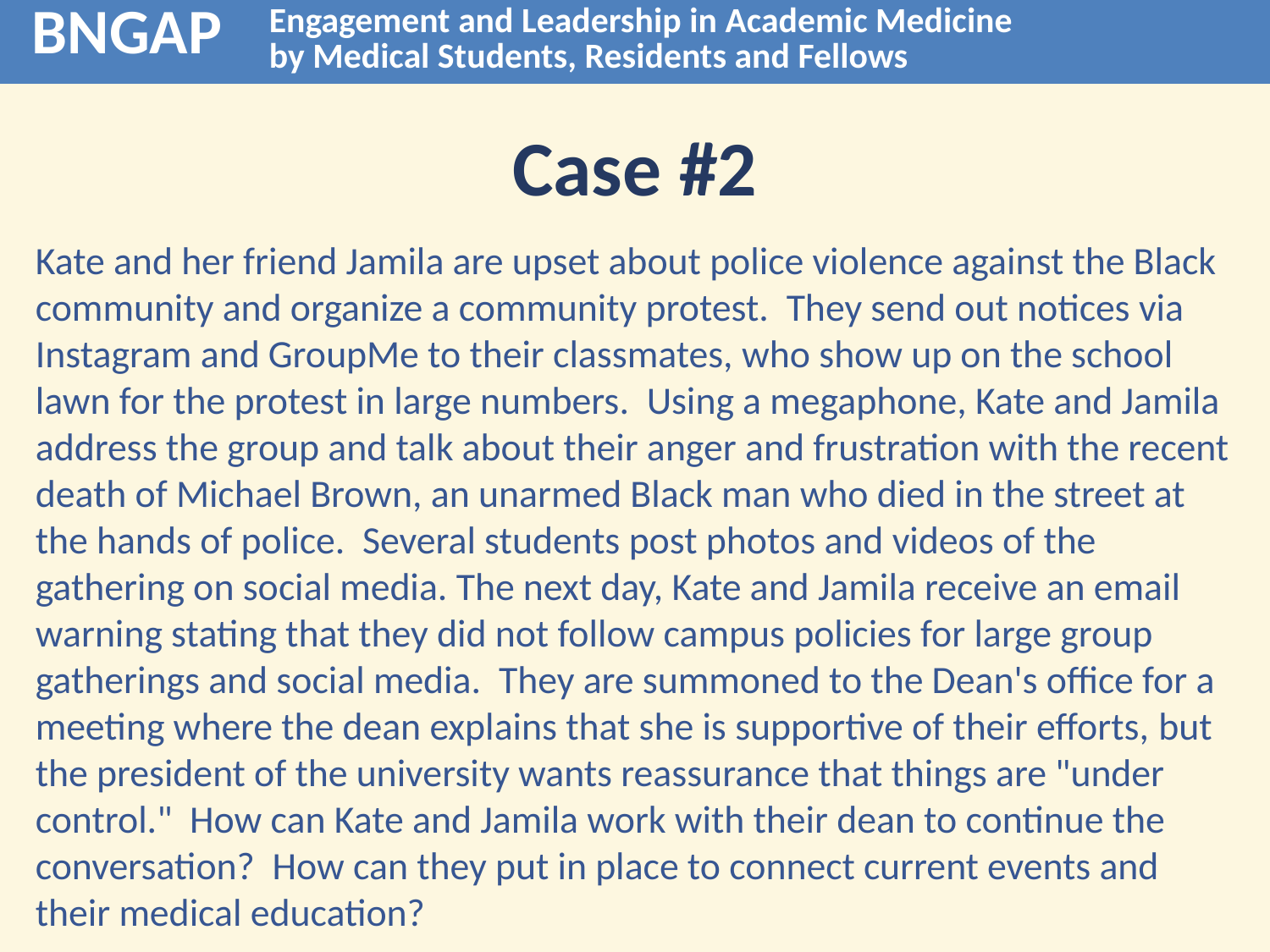

# Case #2
Kate and her friend Jamila are upset about police violence against the Black community and organize a community protest.  They send out notices via Instagram and GroupMe to their classmates, who show up on the school lawn for the protest in large numbers.  Using a megaphone, Kate and Jamila address the group and talk about their anger and frustration with the recent death of Michael Brown, an unarmed Black man who died in the street at the hands of police.  Several students post photos and videos of the gathering on social media. The next day, Kate and Jamila receive an email warning stating that they did not follow campus policies for large group gatherings and social media.  They are summoned to the Dean's office for a meeting where the dean explains that she is supportive of their efforts, but the president of the university wants reassurance that things are "under control."  How can Kate and Jamila work with their dean to continue the conversation?  How can they put in place to connect current events and their medical education?

## Slide 30
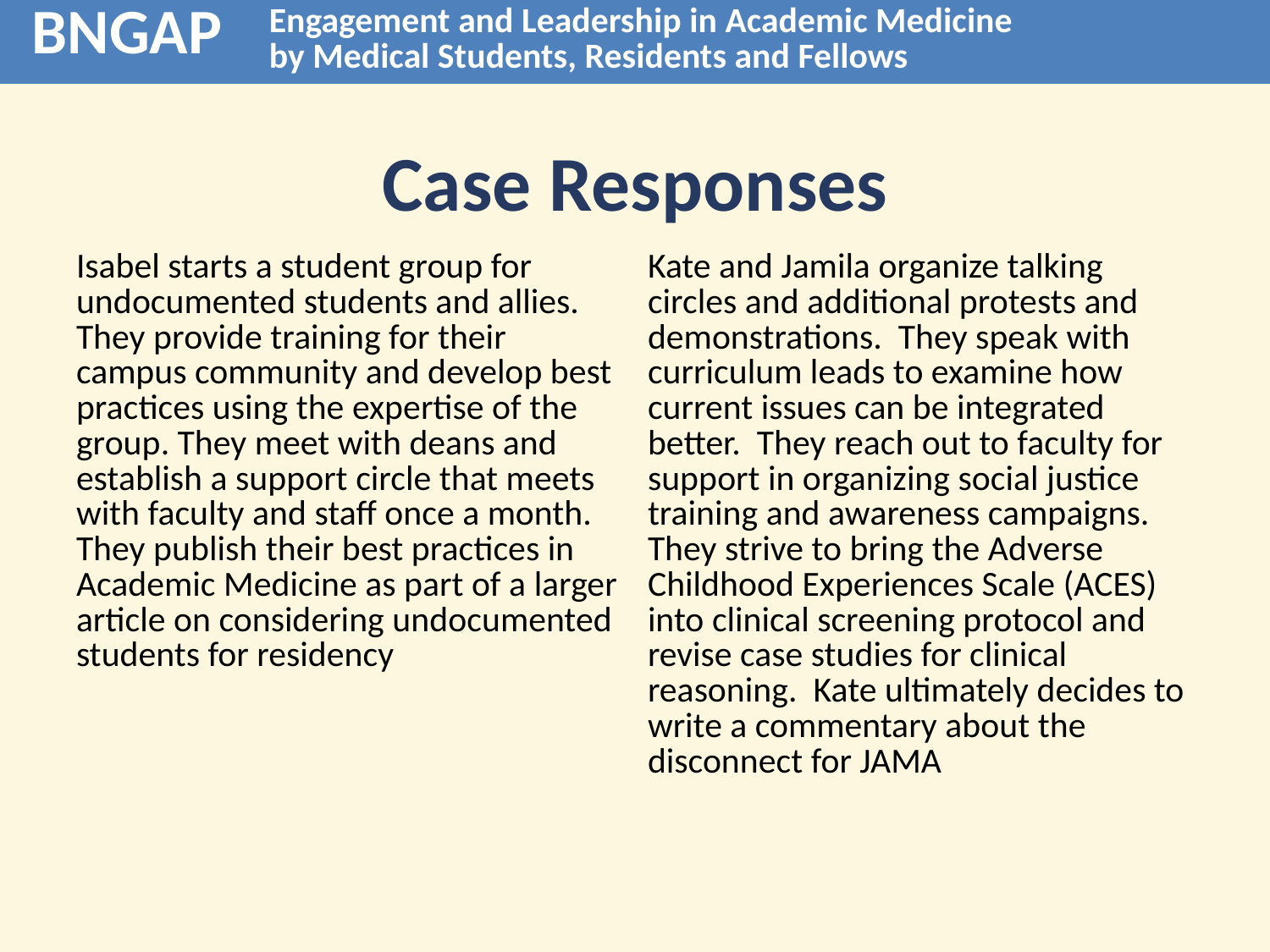

# Case Responses
| Isabel starts a student group for undocumented students and allies. They provide training for their campus community and develop best practices using the expertise of the group. They meet with deans and establish a support circle that meets with faculty and staff once a month. They publish their best practices in Academic Medicine as part of a larger article on considering undocumented students for residency | Kate and Jamila organize talking circles and additional protests and demonstrations.  They speak with curriculum leads to examine how current issues can be integrated better.  They reach out to faculty for support in organizing social justice training and awareness campaigns.  They strive to bring the Adverse Childhood Experiences Scale (ACES) into clinical screening protocol and revise case studies for clinical reasoning.  Kate ultimately decides to write a commentary about the disconnect for JAMA |
| --- | --- |

## Slide 31
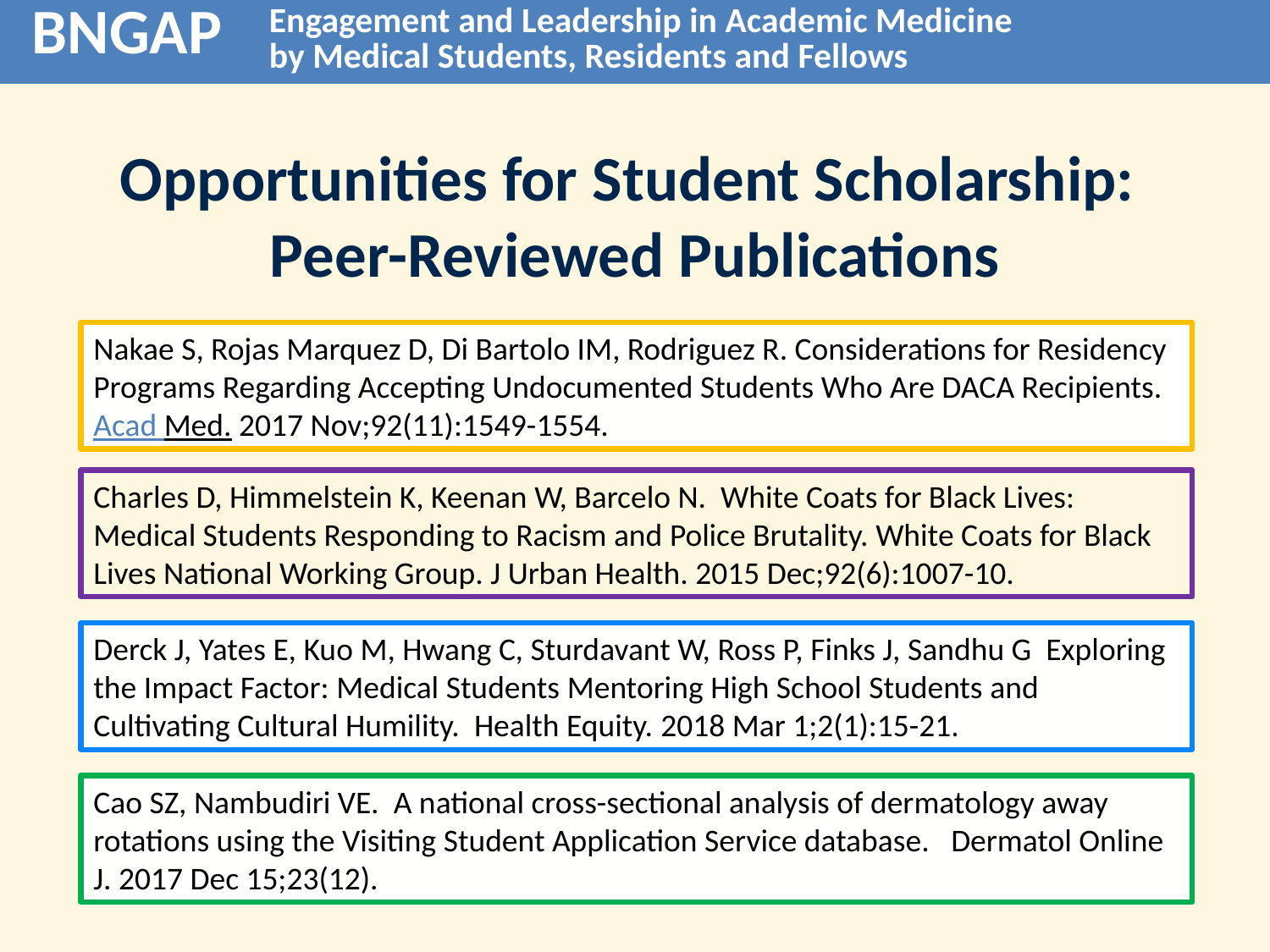

# Opportunities for Student Scholarship: Peer-Reviewed Publications
Nakae S, Rojas Marquez D, Di Bartolo IM, Rodriguez R. Considerations for Residency Programs Regarding Accepting Undocumented Students Who Are DACA Recipients. Acad Med. 2017 Nov;92(11):1549-1554.
Charles D, Himmelstein K, Keenan W, Barcelo N. White Coats for Black Lives: Medical Students Responding to Racism and Police Brutality. White Coats for Black Lives National Working Group. J Urban Health. 2015 Dec;92(6):1007-10.
Derck J, Yates E, Kuo M, Hwang C, Sturdavant W, Ross P, Finks J, Sandhu G Exploring the Impact Factor: Medical Students Mentoring High School Students and Cultivating Cultural Humility. Health Equity. 2018 Mar 1;2(1):15-21.
Cao SZ, Nambudiri VE. A national cross-sectional analysis of dermatology away rotations using the Visiting Student Application Service database. Dermatol Online J. 2017 Dec 15;23(12).

## Slide 32
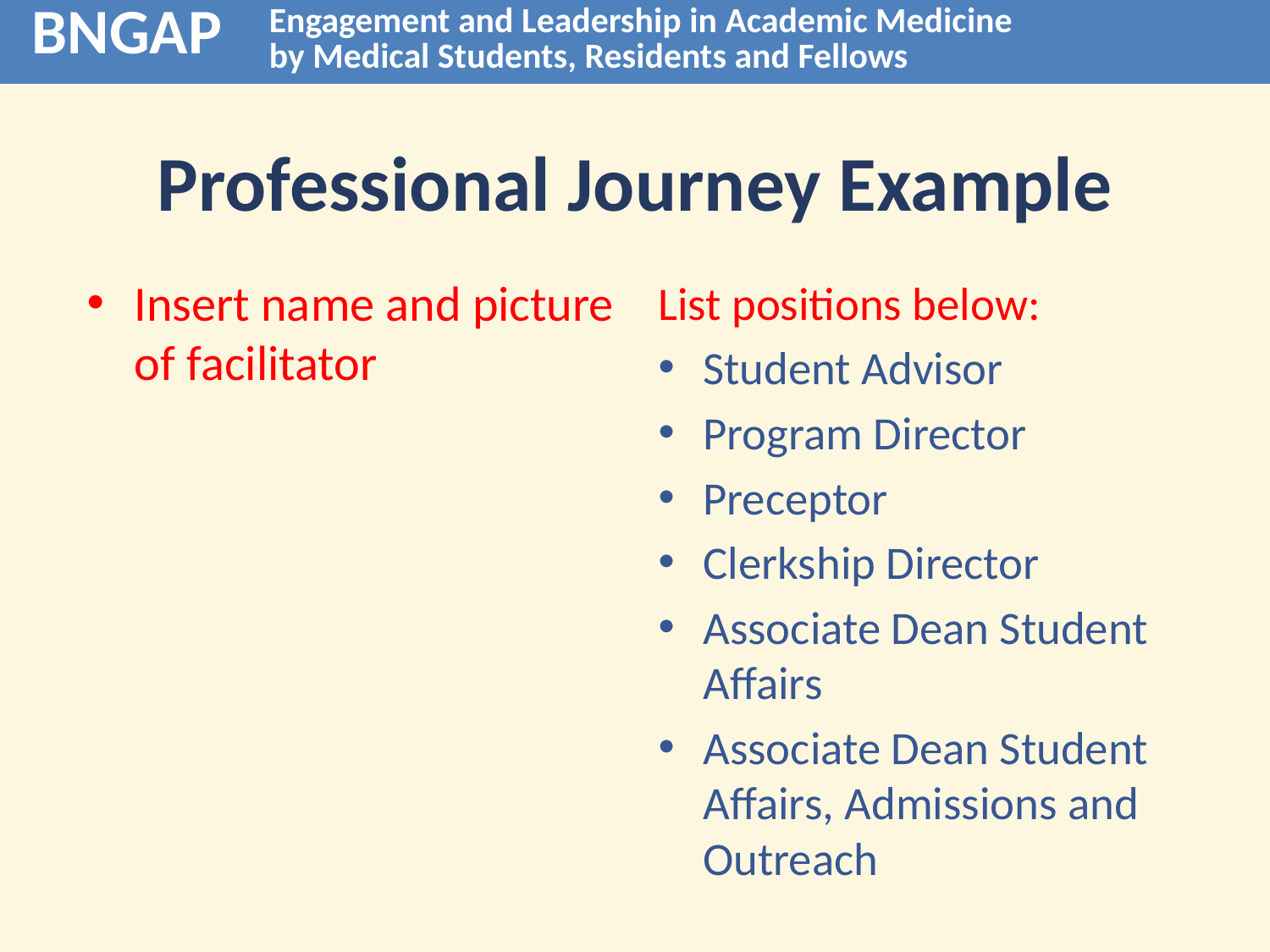

# Professional Journey Example
Insert name and picture of facilitator
List positions below:
Student Advisor
Program Director
Preceptor
Clerkship Director
Associate Dean Student Affairs
Associate Dean Student Affairs, Admissions and Outreach

## Slide 33
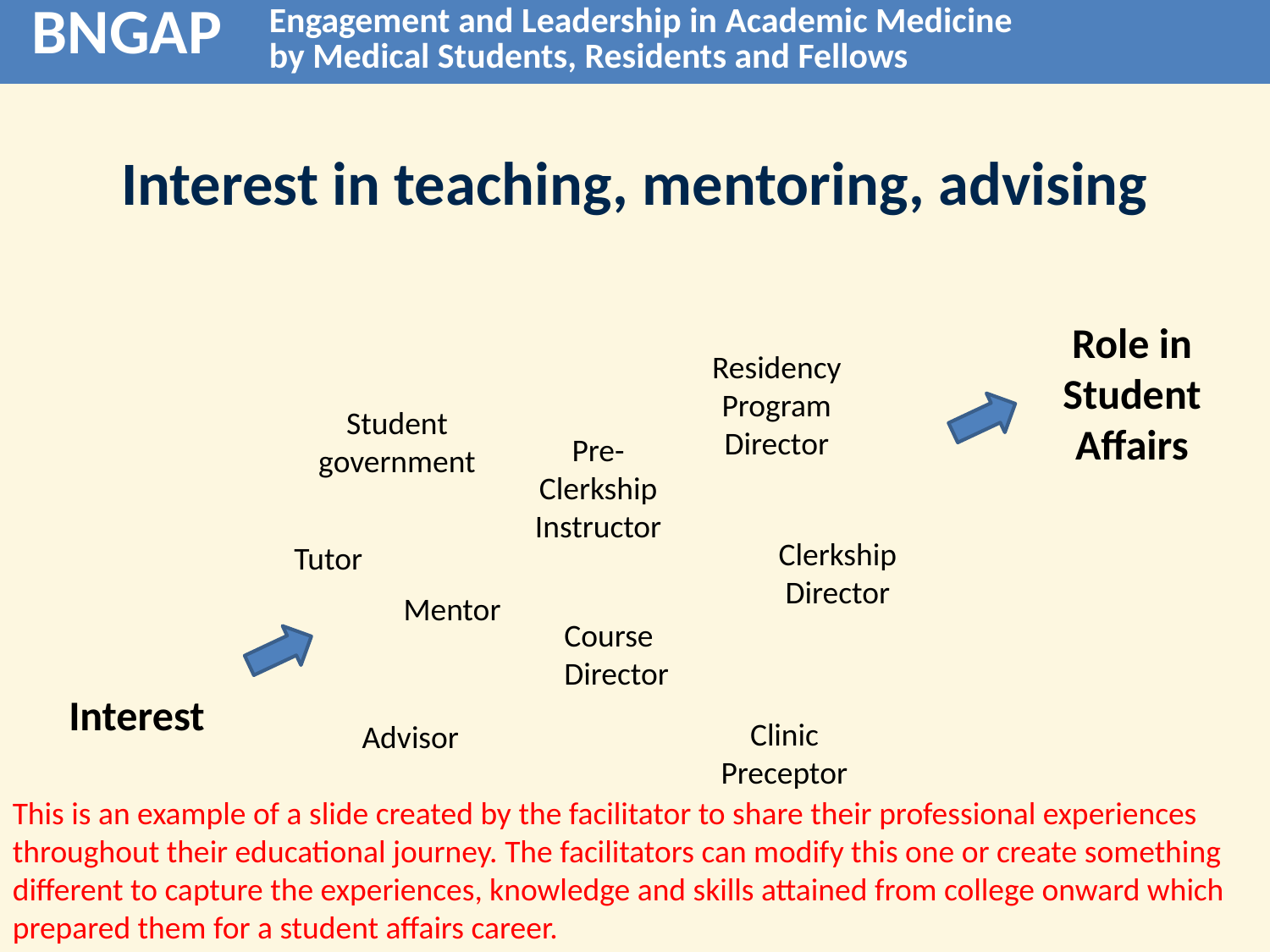

# Interest in teaching, mentoring, advising
Role in Student Affairs
Residency Program Director
Student government
Pre-Clerkship Instructor
Clerkship Director
Tutor
Mentor
Course Director
Interest
Clinic Preceptor
Advisor
This is an example of a slide created by the facilitator to share their professional experiences throughout their educational journey. The facilitators can modify this one or create something different to capture the experiences, knowledge and skills attained from college onward which prepared them for a student affairs career.

## Slide 34
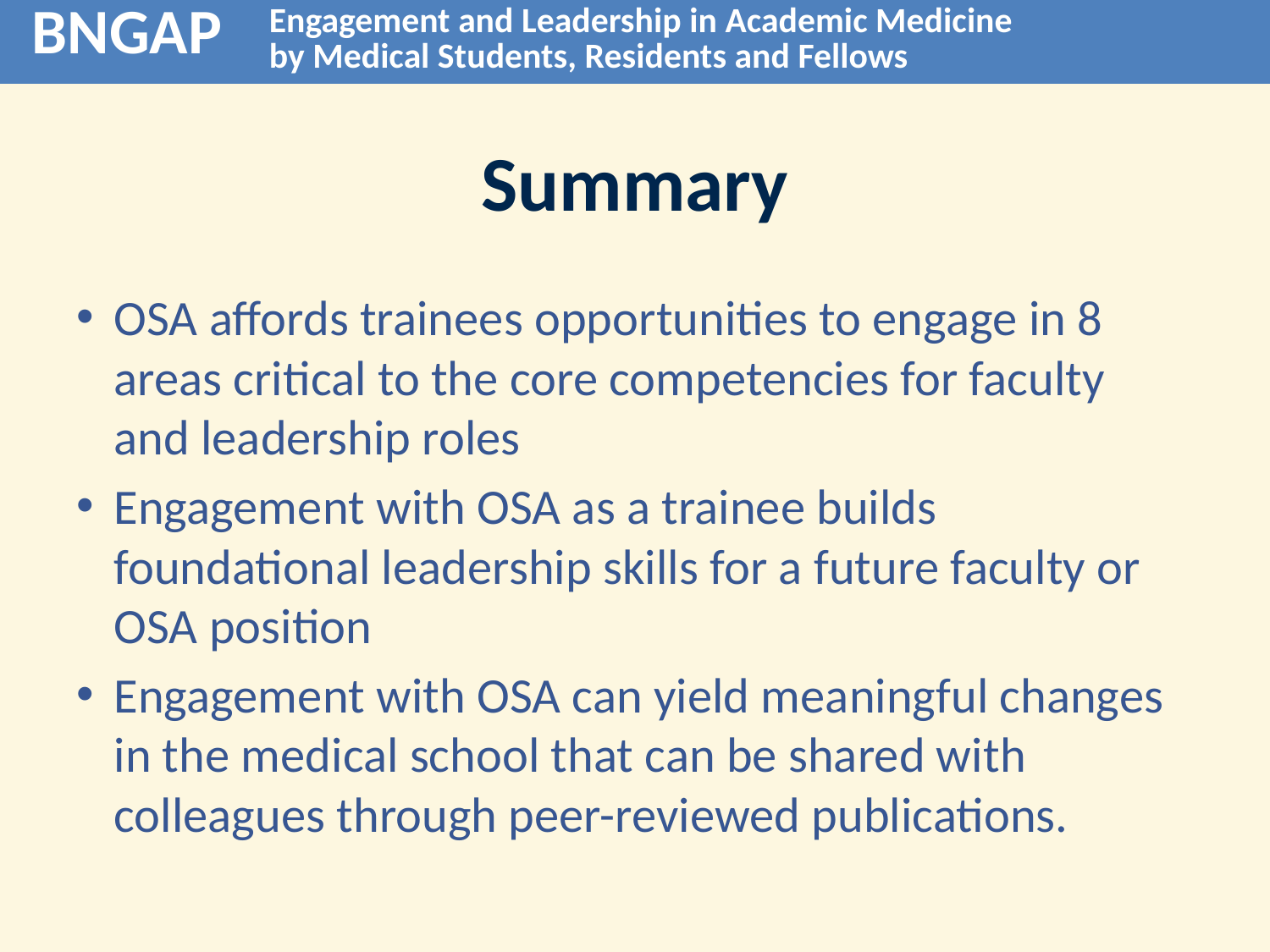

# Summary
OSA affords trainees opportunities to engage in 8 areas critical to the core competencies for faculty and leadership roles
Engagement with OSA as a trainee builds foundational leadership skills for a future faculty or OSA position
Engagement with OSA can yield meaningful changes in the medical school that can be shared with colleagues through peer-reviewed publications.

## Slide 35
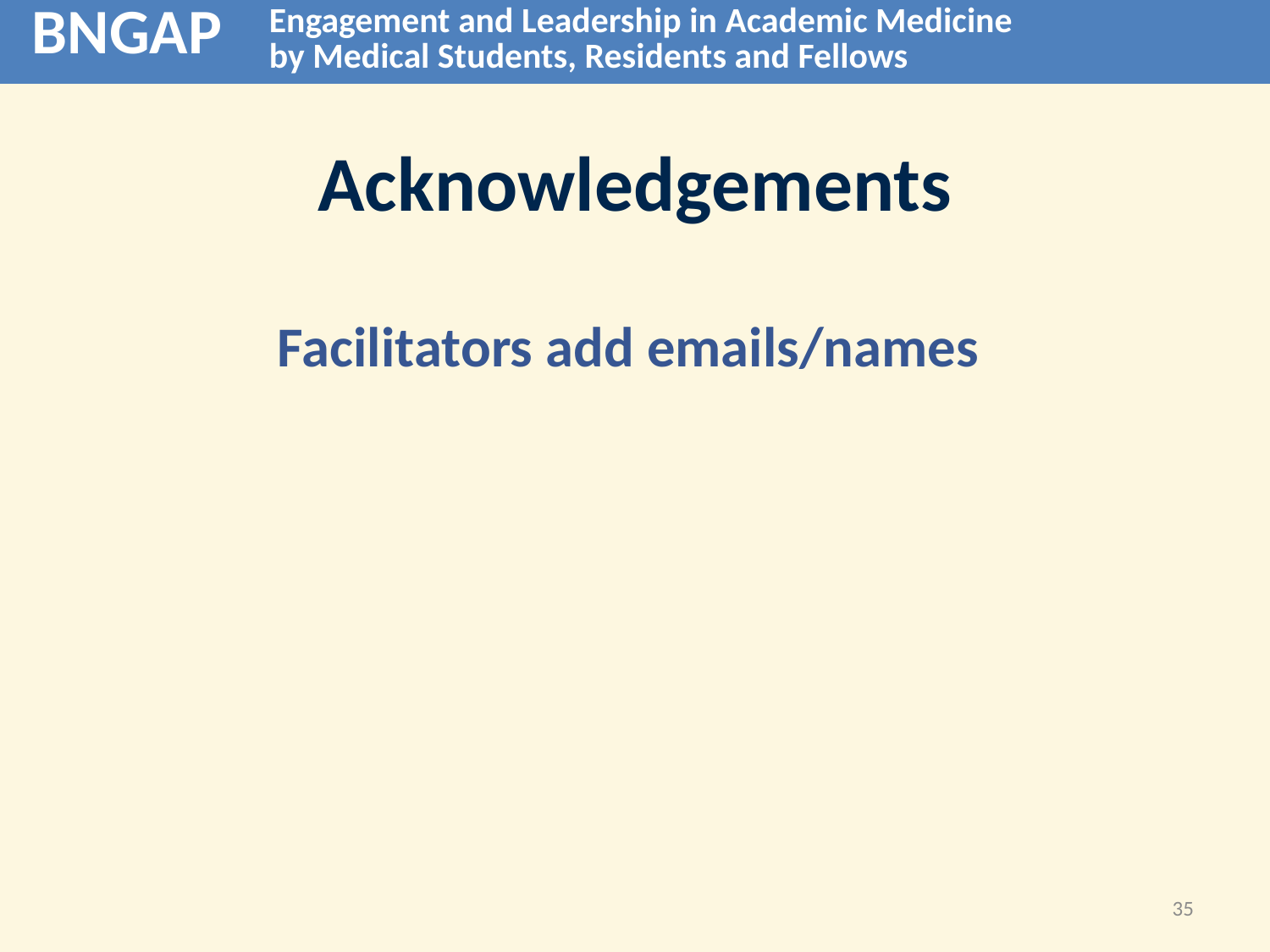

# Acknowledgements
Facilitators add emails/names
35

## Slide 36
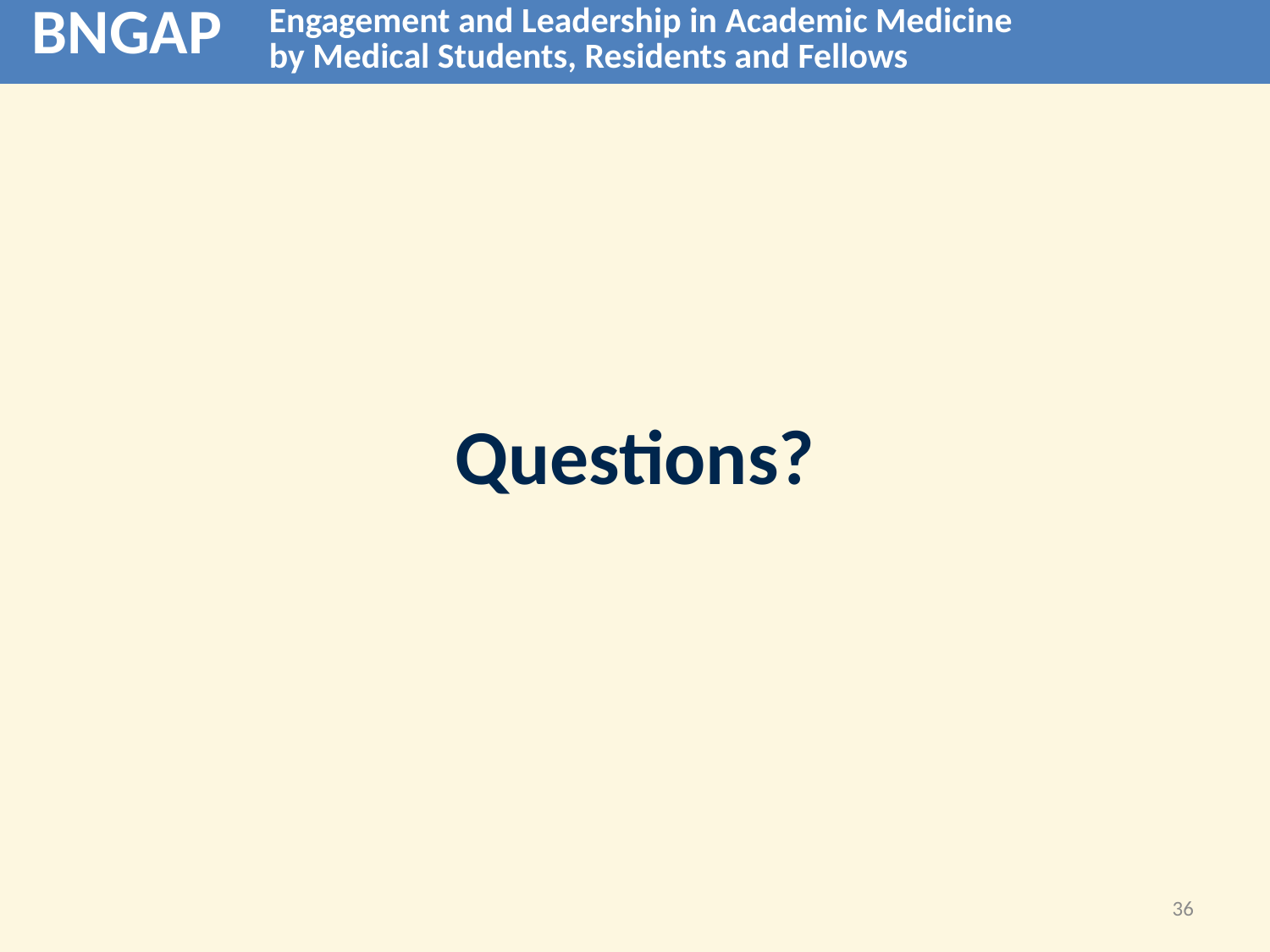

# Questions?
36
