# Supplementary material for: Office of Student Affairs: Engagement and Leadership Opportunities for Medical Students, Residents, and Fellows
Source: MedEdPORTAL. 2021 Feb 5;17:11093. doi: 10.15766/mep_2374-8265.11093 (PMC7880253; doi:10.15766/mep_2374-8265.11093)
Supplement: Supplementary file 1 — OSA Evaluation Forms.docxOSA PowerPoint.pptxOSA Duties Activity.docxOSA Chart.docxOSA Cases.docxOSA Facilitator Guide.docx [file mep_2374-8265.11093-s001.zip › C. OSA Duties Activity.docx]

**Office of Student Affairs Duties Activity**

Provisions needed:

1) large room with wall space or easels where items can be taped or tacked to walls
2) poster markers, 1 per group
3) large labels for the core and collaborative OSA areas (printed signs, written on white board, etc.)
4) 62 sheets of paper (any size) each with a scenario printed on it
5) adhesive to post sheets of paper beneath areas on walls (tacks, tape, etc)

Designate areas around the room for each of the Core and Collaborative OSA areas below. You may write areas on a white board, use a bulletin, use large sticky note pads, etc. Each area should be designated as a separate space where items from the numbered list (below) can be posted individually beneath.

Core OSA areas:

- Career Advising & Development
- Academic Progress
- Diversity & Inclusion
- Wellness
- Recruitment & Admissions
- Student Engagement
- Financial Assistance
- Unit Operations
- Records Management

Collaborative OSA areas:

- Curriculum
- Faculty Development
- Community Partnerships
- Health Services
- Compliance
- Facilities & Space Management

Print the following items for the exercise, using one sheet of paper per item. You will have 62 pieces of paper, each containing one item from the list, for this activity. You may vary the size of the paper according to your group size. A full sheet is recommended so that participants can easily see the items while milling around the room.

1. Meeting with a student to discuss her specialty choice
2. Connecting a student with a faculty mentor to nurture his interest in Psychiatry
3. Arranging a student/faculty specialty mixer for students to learn more about specialty areas
4. Meeting with a student who has failed an exam
5. Attending progress and promotions committee to deliberate a student dismissal
6. Meeting with a student who is asking to change their schedule in order to have more time to study for Step 2CK
7. Meeting with the dean to request money for an LMSA regional conference
8. Attending the Faculty Diversity Committee
9. Meeting with education after the block review revealed biased comments that occurred during lecture
10. Obtaining permission from the dean to support a student protest
11. Organizing a student mental health awareness panel
12. Meeting with a student to check on them following a death in the family
13. Working with campus fitness facility to reduce medical student fees for use
14. Requesting extended hours for student counseling from the campus mental health provider
15. Recruiting prospective applicants at a premedical conference
16. Attending admissions committee meetings as a voting member
17. Providing a training for the admissions committee on unconscious bias and structural racism
18. Attending student leadership council
19. Working with the Pediatrics interest group to plan their annual conference
20. Meeting with rising M4s to plan the Match Day celebration
21. Discussing a professionalism issue with a student who is posting inappropriate material on TikTok
22. Exploring resources for a student who cannot afford the Step 1 fee
23. Organizing a budgeting and money management seminar for medical students
24. Meeting with the scholarship committee to present recommendations for awards for recently admitted students
25. Presenting the annual OSA budget to the Chief Financial Officer
26. Preparing performance reviews for the OSA team members
27. Reviewing quarterly financial statements for the OSA budget
28. Providing a letter of good standing for a student applying for an away rotation
29. Establishing protocol for students to show proof of immunization for clinical activities
30. Certifying students for promotion to subsequent years and graduation
31. Registering students for courses and clerkships
32. Responding to a student who is requesting accommodation for disability
33. Working with students to develop an LGBTQ+ module for the social medicine course
34. Designing and presenting a faculty development module on inclusive pedagogy
35. Writing a letter of recommendation for a student applying for an external scholarship
36. Reviewing student ratings and comments submitted for course/block reviews
37. Attending Medical Education Committee meeting
38. Meeting with a faculty member after a grievance was submitted regarding their unprofessional behavior
39. Obtaining legal and risk management clearance for the student run free clinic
40. Attending the community board of advisers meeting to understand partnership needs
41. Volunteering for the street medicine outreach program
42. Meeting with a local hospital to obtain clearances for the Music in Medicine interest group to do bedside performances
43. Working with student health services to establish reasonable fees for TB testing
44. Meeting with the campus health insurance committee to assess bids for the next insurance provider contract
45. Exploring provider options for transhealth in the area for a new student who needs care
46. Communicating with student about the annual flu shot requirement and collecting proof of immunizations
47. Designing a training module for students on procurement rules and regulations
48. Submitting a revision to the student handbook on the excused absences policy for students who are parents
49. Ensuring proper clinician supervision and HIPAA compliance for the student-run free clinic
50. Compiling the annual report for blood borne pathogens and exposures of medical students at all clinical sites
51. Establishing emergency communication protocols for the OSA team in responding to student emergencies and natural disasters
52. Working with building management to order standing desks for the library
53. Responding to a circuit outage in the student lounge that has resulted in all microwaves not working
54. Helping identify a space on campus for a student-led yoga class
55. Helping a student initiate a leave of absence for a substance use disorder
56. Consulting with the education dean on a student grade appeal
57. Knowledge of FERPA
58. Knowledge of mandatory reporting of sexual harassment and gender violence under Title IX
59. Knowledge of state based laws governing prohibited selection criteria in admissions
60. Revising a CV for a student
61. Helping a student with a residency personal statement
62. Understanding the Americans with Disabilities Act and the school’s Technical Standards for Degree Completion

Divide the room into groups of 2-4 participants and distribute the 62 papers (each containing one item) evenly between groups. Instruct the groups to put their items under the OSA core or collaborative areas where they think they fit best. If they determine their item falls into more than one category, ask them to put a large circle on it before posting it. If they determine that the item is an area for potential student and resident engagement in the OSA, ask them to put a star on it.

When all the items have been posted, debrief the exercise with the group.

Where did you put your items and why?

Which items feel into more than one category?

Which items were ideal for student and resident engagement in OSA leadership?

Did any of the items surprise you as being part of the scope of the OSA?

Did you identify any areas for student engagement that were new to you?
