# Supplementary material for: Office of Student Affairs: Engagement and Leadership Opportunities for Medical Students, Residents, and Fellows
Source: MedEdPORTAL. 2021 Feb 5;17:11093. doi: 10.15766/mep_2374-8265.11093 (PMC7880253; doi:10.15766/mep_2374-8265.11093)
Supplement: Supplementary file 1 — OSA Evaluation Forms.docxOSA PowerPoint.pptxOSA Duties Activity.docxOSA Chart.docxOSA Cases.docxOSA Facilitator Guide.docx [file mep_2374-8265.11093-s001.zip › D. OSA Chart.docx]

APPENDIX C

| **Office of Student Affairs: Engagement and Leadership Opportunities for Medical Students** | Sunny Nakae, PhD, MSW, Senior Associate Dean for Diversity, Equity, Inclusion, and Partnership, California University of Science and Medicine |
| --- | --- |

According to the 2018 AAMC Year Two Questionnaire^1^ approximately 23% of medical students plan to participate in medical school administration during their career. The Office of Student Affairs serves as one avenue for medical students to become engaged and develop competencies to serve as future administrative leaders.

**Role of the Office of Student Affairs:**

To provide comprehensive support for medical students in recruitment, admissions, academic progression, career development, wellness, leadership development, professionalism development, and financial stewardship to ensure student success and graduation.

**Relevance of Medical Students Being Engaged in Student Affairs:**

Students are essential partners in student affairs activities, policies, and processes that play a key role in developing leadership competencies. Medical students are frequently involved in extracurricular and co-curricular activities and leadership endeavors in partnership and coordination with student affairs.  Students can help adapt and design programs to meet student needs while also furthering the mission of the institution. Engagement in student affairs provides the opportunity to learn important skills that are foundational to academic medicine.

| Faculty Leadership Competencies^2^ Achievable Through Admissions-Related Activities | | | |
| --- | --- | --- | --- |
| 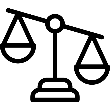  **Self-management:** demonstrates emotional intelligence in encounters with peers, faculty, staff and administration | 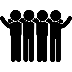  **Working with/developing others:**  leads and coaches peers in student organizations, committees, service endeavors, and support services | | 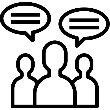 **Communication skills**: effectively presents and expresses ideas, engages in dialogue, and demonstrates strong problem solving and conflict management skills |
| 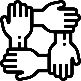 **Teambuilding**: works with peers, faculty, staff and administration to develop and accomplish shared goals | 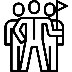 **Leadership:** understands the structure and culture of the institution and effectively navigates and advocates for important issues | 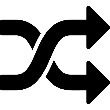 **Leading change:** engages in change management through development of new or revised policies and procedures within institutional parameters | 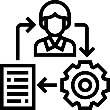 **Business skills:** manages resources strategically while attending to policies and procedures and institutional compliance guidelines |

|  | *Types and year of participation varies by institution. | 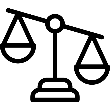 | 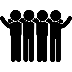 | 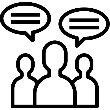 | 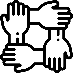 | 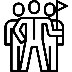 | 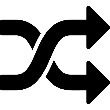 | 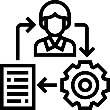 |
| --- | --- | --- | --- | --- | --- | --- | --- | --- |
| 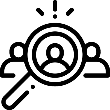 | **Coaching, Tutoring, or Mentoring**  Assists with peer programming aimed at developing student acumen, sharing knowledge, or maintaining wellbeing. Assists with recruitment by fulfilling these roles for prospective students in the pipeline. | **•** | **•** | **•** | **•** |  |  |  |
| 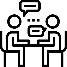 | **Participates in student government or leadership**  Enacts leadership for various student groups centered on identity, interest, community/civic engagement, or career development.  Manages resources, both human and financial, to meet organizational goals. | **•** | **•** | **•** | **•** | **•** | **•** | **•** |
| 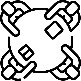 | **Represent Student Interests**  Participates and provides feedback in the enactment and implementation of policies and procedures that directly impact students.  May serve as intermediary between peers and faculty or administration. | **•** | **•** | **•** | **•** | **•** |  |  |
| 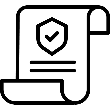 | **Develops New Programs and Events**  Works closely with faculty and administrators to develop new programs and events  in strategic institutional areas such as community service, pipeline programs, learning events, research, etc. |  |  | **•** |  | **•** | **•** | **•** |
| Future  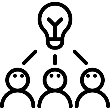 | **Leads as a Faculty Member or Administrator**  Engages in or leads medical student, resident, fellow or faculty development and support programs.  Advocates for comprehensive resources that facilitate success. | **•** | **•** | **•** | **•** | **•** | **•** | **•** |

**Acknowledgements:** We thank FlatIcon for the access and use of their free vector icons **References:**

1. Association of American Medical Colleges. Medical School Year Two Questionnaire 2018 All Schools Summary Report. <https://www.aamc.org/system/files/reports/1/y2q2018report.pdf>. Accessed February 18, 2020.

2. Lucas R, Goldman EF., Scott AR et al. Leadership Development Programs at Academic Health Centers: Results of a National Survey. Academic Medicine. 93(2):229-236, February 2018.
