# Supplementary material for: Office of Student Affairs: Engagement and Leadership Opportunities for Medical Students, Residents, and Fellows
Source: MedEdPORTAL. 2021 Feb 5;17:11093. doi: 10.15766/mep_2374-8265.11093 (PMC7880253; doi:10.15766/mep_2374-8265.11093)
Supplement: Supplementary file 1 — OSA Evaluation Forms.docxOSA PowerPoint.pptxOSA Duties Activity.docxOSA Chart.docxOSA Cases.docxOSA Facilitator Guide.docx [file mep_2374-8265.11093-s001.zip › E. OSA Cases.docx]

**Case Scenarios**

Case 1:

Isabel is a student activist who has worked with undocumented premedical students and medical students through her personal and professional networks.  As an undocumented medical student herself, she has faced challenges as a student due to her immigration status such as getting financial aid, participating in summer internships, and completing her background check.  Although she has a supportive mentor at her school, there are still many instances where she feels that others are not prepared to support her and lack knowledge about undocumented students.  How might Isabel go about improving things for herself and future undocumented students?

- What Student Affairs values or principles apply to this scenario?
- With whom would you strive to collaborate?
- What skills are needed to adapt and respond to stakeholders?
- What is the unique role students can play?
- What scholarly question might you generate from this scenario?

Case 2

Kate and her friend Jamila are upset about police violence against the Black community and organize a community protest.  They send out notices via Instagram and GroupMe to their classmates, who show up on the school lawn for the protest in large numbers.  Using a megaphone, Kate and Jamila address the group and talk about their anger and frustration with the recent death of Michael Brown, an unarmed Black man who died in the street at the hands of police.  Several students post photos and videos of the gathering on social media. The next day, Kate and Jamila receive an email warning stating that they did not follow campus policies for large group gatherings and social media.  They are summoned to the Dean's office for a meeting where the dean explains that she is supportive of their efforts, but the president of the university is concerned.  How can Kate and Jamila work with their dean to continue the conversation?  What can they do to connect current events and their medical education?

- What Student Affairs values or principles apply to this scenario?
- With whom would you strive to collaborate?
- What skills are needed to adapt and respond to stakeholders?
- What is the unique role students can play?
- What scholarly question might you generate from this scenario?
