# Supplementary material for: Office of Student Affairs: Engagement and Leadership Opportunities for Medical Students, Residents, and Fellows
Source: MedEdPORTAL. 2021 Feb 5;17:11093. doi: 10.15766/mep_2374-8265.11093 (PMC7880253; doi:10.15766/mep_2374-8265.11093)
Supplement: Supplementary file 1 — OSA Evaluation Forms.docxOSA PowerPoint.pptxOSA Duties Activity.docxOSA Chart.docxOSA Cases.docxOSA Facilitator Guide.docx [file mep_2374-8265.11093-s001.zip › F. OSA Facilitator Guide.docx]

**Office of Student Affairs: Engagement and Leadership Opportunities for Medical Students, Residents, and Fellows**

**Facilitator’s Guide**

**Purpose of Module:**

This module is meant to be an educational module that provides understanding of the roles and responsibilities of the Office of Student Affairs. This workshop provides an overview of the functions of the Office of Student Affairs. The learners will get an understanding of the various roles medical education leaders can have within student affairs and the collaborating offices. This module will also give an overview of learner engagement opportunities through the Office of Student Affairs. This module may be used as a stand-alone presentation or as a part of a larger curriculum to promote the engagement and leadership of diverse trainees at academic health centers.

Learning Objectives:

1. Describe the roles and responsibilities of the Office of Student Affairs (OSA)
2. Define guiding principles, skills, and behaviors required of student affairs professionals
3. Describe leadership opportunities for medical students, residents, and fellows through the OSA
4. Link student engagement opportunities through the OSA to core competencies for leadership in academic medicine roles

Companion materials for this session:

1. PowerPoint Presentation
2. Handout 1 – Office of Student Affairs Duties Activity
3. Handout 2 - OSA Leadership & Engagement Heuristic

**Slide 1**- Title

**Slide 2** – List facilitator(s) disclosures.

**Slide 3** – Learning objectives. Read these to the students.

**Slide 4** – Session overview. Give learners an overview of the module by going over the session overview.

**Slide 5** – Opening reflections. Read the questions on the slide and ask learners to self-reflect. Ask for one or two volunteers to share their reflections with the group.

**Slide 6 –** Host institution information. The facilitators should provide institutional context for their student affairs team(s) by outlining the organizational chart, reporting lines, and duties of their local student affairs office. If more than one institution is presenting or hosting, add more slides.

**Slide 7** – This slide illustrates an example day for a student affairs leader. It can be a combination of institutional level meetings as part of the leadership team, participating in committees, and advising students. If the facilitator would like to replace this with a screen shot of their own schedule for a day/week, they may.

**Slide 8** – This model illustrates the American Association of Medical Colleges (AAMC) Group on Student Affairs Professional Development Initiative framework. All professionals in these different areas demonstrate common attributes such as leadership, organizational acumen, relationships, and service. Some schools may have all 8 of these areas in their student affairs office, and others may have them split between university and medical school or other offices within the medical school. Facilitators can go to the site where the PDI is online and show it live: <https://www.aamc.org/professional-development/affinity-groups/gsa/professional-development-initiative>

**Slide 9** – This slide reviews the core areas for student affairs professionals.

**Slide 10** – This slide shows the other areas of medical education that the OSA may collaborate with on the leadership team.

**Slide 11** – Explain the instructions for the interactive activity. Divide the room into groups of 2-4 participants and distribute the 62 papers (each containing one item) evenly between groups. Instruct the groups to put their items under the OSA core or collaborative areas where they think they fit best. If they determine their item falls into more than one category, ask them to put a large circle on it before posting it. If they determine that the item is an area for potential student and resident engagement in the OSA, ask them to put a star on it. After they have completed the activity, bring the group back together to debrief. Ask a volunteer from each group to report the answers to the following questions:

- Where did you put your items and why?
- Which items feel into more than one category?
- Which items were ideal for student and resident engagement in OSA leadership?
- Did any of the items surprise you as being part of the scope of the OSA?
- Did you identify any areas for student engagement that were new to you?

Facilitators can anticipate that almost all the scenarios will have overlap between core and collaborative areas, or both. All scenarios can contain a star indicating an opportunity for trainee engagement. If a scenario does not contain a star, challenge to group to consider how trainees could become involved in, inform, or assist with the issue. One of the important takeaways is the incredibly broad scope of student affair practice in medical education, thus helping participants identify opportunities for engagement. Probe participants after the exercise if there were any scenarios that were familiar to them, and if they had previously considered personally engaging. Why? Why not? As a result of the workshop would they consider engaging in the future?

**Slide 12** – Review the core values that inform the work done in student affairs. Provide examples of how the values are demonstrated in practice. Adaptability - SA professionals must be able to respond to changes in the educational environment on micro and maco levels. The MD and DO programs merging into single accreditation is a macro change. The curriculum changing at your school is a micro change. Advocacy - SA leaders engage in advocacy in order to ensure that students have an equitable educational experience. This might mean navigating the university’s disability accommodations process or lobbying for childcare on campus. Advocacy happens when there is a disconnect between policy and need. It means both helping people and changing policy when necessary. Diversity & Inclusion - SA professionals are called upon to ensure that all students thrive. The means focusing on areas of diversity and inclusion in practice such as supporting vulnerable or underrepresented students or groups and working to address racism, sexism and inequality in education. Excellence - In all we do SA professionals aim for continuous improvement and excellence. SA leaders worked with the NRMP and ERAS leaders to create the SOAP program when they recognized that a better system was needed to assist students who went unmatched. Professionalism - SA leaders are expected to model and demonstrate professionalism in all they do. As mentors and leaders, students look to SA professionals as role models. Service - SA leaders are there to serve students. Their goal is to ensure that every student thrives and reaches their full potential in medical education. SA professionals often work around the clock ensuring student wellbeing.

**Slide 13 –** Review the guiding principles that inform the work along with core values in student affairs.

**Slide 14** – This graphic depicts all the areas that student affairs professionals collaborate with. They can be involved with medical students from recruitment to graduation.

**Slide 15** – Student Affairs professionals may be involved with recruitment or collaborate with office that does recruitment.

**Slide 16** – Student Affairs professionals are actively involved with aspects related to matriculation and collaborate with offices that are also involved with matriculation of medical students. This maybe be for orientation, clinical compliance and onboarding, or pre-matriculation programs.

**Slide 17** – Student Affairs professionals are actively involved with support of students during their academic progression. They are often involved with policies and programs to ensure successful academic progression for all students. They do this by attending meetings as voting members or serving as student advocates.

**Slide 18** – Student Affairs professionals are actively involved with support of students in their career development. They often empower and guide students in setting their professional goals. SA leaders are often responsible for delivering the careers in medicine curriculum to their students.

**Slide 19** – Student Affairs professionals are actively involved with students throughout the match process and graduation. This includes supporting students through the Match process and the SOAP.

**Slide 20** – This slide gives examples of faculty in Student Affairs roles. You can insert your own colleagues here if you wish. For each role, highlight their biographies and point out that there is diversity in student affairs roles. To be inclusive showcase individuals of different races and ethnicities, genders, and sexual orientations.

**Slide 21** – This slide points out some of the different roles that can be found in the Office of Student Affairs.

**Slide 22** – Now that we have reviewed 8 areas overseen by OSA, ask learners “Which role(s) appeal to you?” and “In which areas can medical students and residents become engaged?

Trainees have the opportunity to become engaged on all areas to refine their knowledge- and skill-base.

**Slide 23** – This is a heuristic that concisely illustrates the leadership competencies achievable through engaging within the Office of Student Affairs.

This heuristic is based on the work by Lucas R, Goldman E, Scott A, Dandar V. Leadership Development Programs in Academic Medical Centers: Results of a National Survey. *Academic Medicine 2018;*93:229-236. This publication describes efforts by professional development/faculty affairs dean towards developing leadership skills among their faculty. The article describes 7 leadership competencies of importance for faculty to develop as leaders. This diagram lists the seven competencies and provides examples of how trainees can develop these competencies through a variety of familiar activities.

**Slide 24** – This chart provides additional specific examples of how medical students and residents can become engaged in leadership opportunities inside and outside of their medical school.

**Slide 25** - AAMC Organization of Student Representatives (OSR) is a good way for students to have leadership opportunities. This lists some of the available opportunities through OSR. The AAMC Organization of Resident Representatives is an opportunity for residents to serve as leaders within the Council for Faculty and Academic Societies.

**Slide 26** – Ask participants to consider the benefits of leadership in student affairs as trainees. How would engagement facilitate building networks with other future SA professionals? How would engagement create opportunities for advocacy, mentorship, and scholarship?

**Slide 27 –** Transition slide – case scenarios

**Slide 28** - Ask for a volunteer to read Case #1. Ask for volunteers to answer the following questions:

- What Student Affairs values or principles apply to this scenario?
  - Students may respond with: diversity, equity, inclusion, wellness, student support, retention, quality improvement
- With whom would you strive to collaborate?
  - Students may respond with: financial aid office, registrar’s office, medical student council/leadership, admissions office, student affairs dean
- What skills are needed to adapt and respond to stakeholders? (refer back to slide 23)
  - Students may respond with: working with and developing others, leading change, teambuilding, leadership, business skills, etc. For each skill area, ask them to provide an example or approach
- What is the unique role students can play?
  - Students may respond with: gathering information from classmates, using the power of story or experiences to educate and influence stakeholders, providing background information and research on the issue, helping stakeholders identify the gaps
- What scholarly question might you generate from this scenario?
  - Students might respond with: What are the experiences of undocumented students in medicine? How many schools have undocumented medical students? What are schools doing to adapt their policies and procedures to serve undocumented students? What are the barriers to inclusion for undocumented students?

**Slide 29** - Ask for a volunteer to read Case #2. Ask for volunteers to answer the following questions:

- What Student Affairs values or principles apply to this scenario?
  - Students may respond with: diversity, equity, inclusion, freedom of speech, freedom of assembly, student support
- With whom would you strive to collaborate?
  - Students may respond with: deans office, university leadership, government relations person, community organizations, faculty mentors, student leaders
- What skills are needed to adapt and respond to stakeholders? (refer to slide 23)
  - Students may respond with: self-management, communication skills, leadership, leading change, etc. For each skill area, encourage examples.
- What is the unique role students can play?
  - Students may respond with: speaking out more boldly than faculty, working together to express their views even if the school doesn’t share them, sharing the impact of racism on them, helping stakeholders understand their experiences in medical education
- What scholarly question might you generate from this scenario?
  - How is racism presented or studied in medicine? What role should current events play in medical education? How have medical schools responded to racism or trauma in the community?

**Slide 30** – This slide summarizes potential responses from the cases discussed in the preceding slides.

Case 1 - Isabel starts a student group for undocumented students and allies.  They provide training for their campus community and develop best practices using the expertise of the group. They meet with deans and establish a support circle that meets with faculty and staff once a month.  They publish their best practices in Academic Medicine as part of a larger article on considering undocumented students for residency - Nakae S, Rojas Marquez D, Di Bartolo IM, Rodriguez R. Considerations for Residency Programs Regarding Accepting Undocumented Students Who Are DACA Recipients. [Acad Med.](https://www.ncbi.nlm.nih.gov/pubmed/28562450) 2017 Nov;92(11):1549-1554.

Case 2 - Kate and Jamila organize dialogue circles and additional protests and demonstrations.  They speak with curriculum leads to examine how current issues can be integrated better.  They reach out to faculty for support in organizing social justice training and awareness campaigns.  They strive to bring the Adverse Childhood Experiences Scale (ACES) into clinical screening protocol and revise case studies for clinical reasoning.  Kate ultimately decides to write a commentary about the disconnect for JAMA. - Charles D, Himmelstein K, Keenan W, Barcelo N. White Coats for Black Lives: Medical Students Responding to Racism and Police Brutality. White Coats for Black Lives National Working Group. J Urban Health. 2015 Dec;92(6):1007-10.

**Slide 31** - Students should strive for scholarship with their leadership opportunities. This is an example of student scholarship that resulted from leadership and engagement.

**Slide 32-33** – Time permitting the facilitators can modify these slides to share their journey to working within the Office of Student Affairs. Slide 32 is an example of a slide created by the facilitator to share their professional experiences throughout their educational journey. The facilitators can modify this one or create something different to capture the experiences, knowledge and skills attained from college onward which prepared them for a student affairs career.

**Slide 34** – Summary slide. Review the takeaways with attendees and follow up on any discussions related to linking faculty leadership competencies with student affairs engagement.

**Slide 35** - acknowledgements

**Slide 36** - questions
